# How a diverse research ecosystem has generated new rehabilitation technologies: Review of NIDILRR’s Rehabilitation Engineering Research Centers

**DOI:** 10.1186/s12984-017-0321-3

**Published:** 2017-11-06

**Authors:** David J. Reinkensmeyer, Sarah Blackstone, Cathy Bodine, John Brabyn, David Brienza, Kevin Caves, Frank DeRuyter, Edmund Durfee, Stefania Fatone, Geoff Fernie, Steven Gard, Patricia Karg, Todd A. Kuiken, Gerald F. Harris, Mike Jones, Yue Li, Jordana Maisel, Michael McCue, Michelle A. Meade, Helena Mitchell, Tracy L. Mitzner, James L. Patton, Philip S. Requejo, James H. Rimmer, Wendy A. Rogers, W. Zev Rymer, Jon A. Sanford, Lawrence Schneider, Levin Sliker, Stephen Sprigle, Aaron Steinfeld, Edward Steinfeld, Gregg Vanderheiden, Carolee Winstein, Li-Qun Zhang, Thomas Corfman

**Affiliations:** 1 University of California at Irvine, Irvine, USA; 2 Augmentative Communication Inc., Monterey, USA; 3 University of Colorado, Denver, USA; 4 The Smith-Kettlewell Eye Research Institute, San Francesco, USA; 5 University of Pittsburgh, Pittsburgh, USA; 6 Duke University Medical Center, Durham, USA; 7 University of Michigan, Ann Arbor, USA; 8 Northwestern University Prosthetics-Orthotics Center, Evanston, USA; 9 Toronto Rehabilitation Institute, Toronto, Canada; 10 Northwestern University, Evanston, USA; 11 Marquette University, Marquette, USA; 12 Shepherd Center, Atlanta, USA; 13 University at Buffalo IDeA Center, Buffalo, USA; 14 Georgia Institute of Technology, Atlanta, Georgia USA; 15 Rehabilitation Institute of Chicago, University of Illinois at Chicago, Chicago, USA; 16 Rancho Los Amigos National Rehabilitation Center, Downey, USA; 17 Lakeshore FoundationUniversity of Alabama-Birmingham, Birmingham, USA; 18 Rehabilitation Institute of Chicago, Chicago, USA; 19 Robotics Institute, Carnegie Mellon University, Pittsburgh, USA; 20 Trace Center, University of Maryland, College Park, Baltimore, USA; 21 University of Southern California, Los Angeles, USA; 22 National Institute on Disability, Independent Living, and Rehabilitation Research, Washington, DC, USA

**Keywords:** Rehabilitation engineering, Disability, Technology

## Abstract

Over 50 million United States citizens (1 in 6 people in the US) have a developmental, acquired, or degenerative disability. The average US citizen can expect to live 20% of his or her life with a disability. Rehabilitation technologies play a major role in improving the quality of life for people with a disability, yet widespread and highly challenging needs remain. Within the US, a major effort aimed at the creation and evaluation of rehabilitation technology has been the Rehabilitation Engineering Research Centers (RERCs) sponsored by the National Institute on Disability, Independent Living, and Rehabilitation Research. As envisioned at their conception by a panel of the National Academy of Science in 1970, these centers were intended to take a “total approach to rehabilitation”, combining medicine, engineering, and related science, to improve the quality of life of individuals with a disability. Here, we review the scope, achievements, and ongoing projects of an unbiased sample of 19 currently active or recently terminated RERCs. Specifically, for each center, we briefly explain the needs it targets, summarize key historical advances, identify emerging innovations, and consider future directions. Our assessment from this review is that the RERC program indeed involves a multidisciplinary approach, with 36 professional fields involved, although 70% of research and development staff are in engineering fields, 23% in clinical fields, and only 7% in basic science fields; significantly, 11% of the professional staff have a disability related to their research. We observe that the RERC program has substantially diversified the scope of its work since the 1970’s, addressing more types of disabilities using more technologies, and, in particular, often now focusing on information technologies. RERC work also now often views users as integrated into an interdependent society through technologies that both people with and without disabilities co-use (such as the internet, wireless communication, and architecture). In addition, RERC research has evolved to view users as able at improving outcomes through learning, exercise, and plasticity (rather than being static), which can be optimally timed. We provide examples of rehabilitation technology innovation produced by the RERCs that illustrate this increasingly diversifying scope and evolving perspective. We conclude by discussing growth opportunities and possible future directions of the RERC program.

## Background

Disabilities cause complex problems in society often unique to each person. A physical disability can limit a person’s ability to access buildings and other facilities, drive, use public transportation, or obtain the health benefits of regular exercise. Blindness can limit a person’s ability to interpret images or navigate the environment. Disabilities in speaking or writing ability may limit the effectiveness of communication. Cognitive disabilities can alter a person’s employment opportunities. In total, a substantial fraction of the world’s population – at least 1 in 6 people – face these individualized problems that combine to create major societal impacts, including limited participation. Further, the average person in the United States can expect to live 20% of his or her life with disability, with the rate of disability increasing seven-fold by age 65 [1].

In light of these complex, pervasive issues, the field of rehabilitation engineering asks, “How can technology help?” Answering this question is also complex, as it often requires the convergence of multiple engineering and design fields (mechanical, electrical, materials, and civil engineering, architecture and industrial design, information and computer science) with clinical fields (rehabilitation medicine, orthopedic surgery, neurology, prosthetics and orthotics, physical, occupational, and speech therapy, rehabilitation psychology) and scientific fields (neuroscience, neuropsychology, biomechanics, motor control, physiology, biology). Shaping of policy, generation of new standards, and education of consumers play important roles as well.

In the US, a unique research center structure was developed to try to facilitate this convergence of fields. In the 1970’s the conceptual model of a Rehabilitation Engineering Center (REC), focusing engineering and clinical expertise on particular problems associated with disability, was first tested. The first objective of the nascent REC’s, defined at a meeting held by the Committee on Prosthetic Research and Development of the National Academy of Sciences, was “to improve the quality of life of the physically handicapped through a total approach to rehabilitation, combining medicine, engineering, and related science” [2]. This objective became a working definition of Rehabilitation Engineering [2].

The first five centers focused on topics including functional electrical stimulation, powered orthoses, neuromuscular control, the effects of pressure on tissue, prosthetics, sensory feedback, quantification of human performance, total joint replacement, and control systems for powered wheelchairs and the environment [2]. The first two RECs were funded by the Department of Health, Education, and Welfare in 1971 at Rancho Los Amigos Medical Center in Downey, CA, and Moss Rehabilitation Hospital in Philadelphia. Three more were added the following year at the Texas Institute for Rehabilitation and Research in Houston, Northwestern University/the Rehabilitation Institute of Chicago, and the Children’s Hospital Center in Boston, involving researchers from Harvard and the Massachusetts Institute of Technology [3]. The Rehabilitation Act of 1973 formally defined REC’s and mandated that 25 percent of research funding under the Act go to them [2]. The establishment of these centers was stimulated by “the polio epidemic, thalidomide tragedy and the Vietnam War, as well as the disability movement of the early 70s with its demands for independence, integration and employment opportunities” [3].

After the initial establishment of these RECs, the governmental funding agency evolved into the National Institute on Disability and Rehabilitation Research (NIDRR, a part of the U.S. Department of Education), and now is the National Institute on Disability, Independent Living, and Rehabilitation Research (NIDILRR, a part of the U.S. Department of Health and Human Services. Today, as we describe below, the RERC’s study a diverse set of technologies and their use by people with a disability, including human-computer interaction, mobile computing, wearable sensors and actuators, robotics, computer gaming, motion capture, wheeled mobility, exoskeletons, lightweight materials, building and transportation technology, biomechanical modeling, and implantable technologies. For this review, we invited all RERCs that were actively reporting to NIDILRR at the onset of this review project in 2015, and had not begun in the last two years, to participate. These were centers that were funded (new or renewal) in the period 2008-2013, except the RERC Wheelchair Transportation Safety, which was funded from 2001-2011. Two of the RERCs did not respond (see Table 1). For each center, we asked it to describe the user needs it targets, summarize key advances that it had made, and identify emerging innovations and opportunities. By reviewing the scope of rehabilitation engineering research through the lens of the RERCs, our goal was to better understand the evolving nature and demands of rehabilitation technology development, as well as the influence of a multidisciplinary structure, like the RERCs, in shaping the producing of such technology. We also performed an analysis of how multidisciplinary the current RERCs actually are (see Table 3), and asked the directors to critique and suggest future directions for the RERC program.

**Table 1:** Rehabilitation Engineering Research Centers described in this review. Shown are the dates each center was funded (possibly including a no-cost extension period), and the lead institution for the center. All RERCs that were actively reporting to NIDILRR at the onset of this project in 2015, and had not begun in the last two years, were invited to participate in this review. These were centers that were funded (new or renewal) in the period 2008-2013, except the RERC Wheelchair Transportation Safety, which was funded from 2001-2011. Two of the RERCs meeting these criteria did not respond to the invitation (RERC on Telecommunications Access at Univ. Wisconsin – Madison and RERC on Hearing Enhancement at Gallaudet University). Note that some of the RERCs have a history of renewal, and thus drew on a longer time period to provide an overview of their accomplishments in the main text

*Mobility*	
1.	Accessible public transportation (2008-2018, Carnegie Mellon University)
2.	Manipulation and mobility (2013-2017, Rehabilitation Institute of Chicago)
3.	Prosthetics and orthotics (1983-2014, Northwestern University)
4.	Technology for children with orthopedic disabilities (2010-2016, Marquette University)
5.	Universal design and the built environment (1999-2019, The State University of New York at Buffalo)
6.	Wheeled mobility and seating (2003-2015, Georgia Institute of Technology)
7.	Wheelchair transportation safety (2001-2011, University of Pittsburgh then University of Michigan)
*Communication and cognition*	
1.	Augmentative and alternative communication (2008-2014, Duke University)
2.	Cognitive technologies (2004-2019, University of Colorado)
3.	Low vision, blindness and multisensory loss (2006-2020, The Smith-Kettlewell Eye Research Institute)
4.	Mobile technology to support health self-management in adolescents with disabilities (2013-2017, University of Michigan)
5.	Technology for successful aging with a disability (2013-2017, Georgia Tech Research Corporation)
6.	Universal interface and information technology access (2003-2017, University of Maryland- College Park – moved from University of Wisconsin-Madison in 2016)
7.	Wireless technologies (2001-2020, Georgia Institute of Technology)
*Rehabilitation Therapy and Exercise*	
1.	Interactive exercise technologies and exercise physiology for people with disabilities (2002- 2016, University of Alabama at Birmingham – moved from University of Illinois at Chicago in 2011)
2.	Rehabilitation robotics (2002-2016, Rehabilitation Institute of Chicago)
3.	Optimizing participation through technology (2008-2013, University of Southern California)
4.	Telerehabilitation (2004-2014, University of Pittsburgh)
5.	Timing investigation dosage implementation (2013-2017, Rehabilitation Institute of Chicago)

NIDILRR provides funding for each RERC for 5 years. At the end of the five year period, if NIDILRR chooses to advertise the specific Center opportunity topic again, then there is an open competition to win the new center. NIDILRR also sometimes announce open calls for new center ideas. Since 1984, 129 RERC’s have been funded, some through multiple grant cycles, with many others starting new by winning a priority renewal competition or by addressing new priorities. Table 1 provides the start date for each active/recently closed center reviewed here. The summaries of the newer centers necessarily focus on their emerging contributions. Others have existed for decades and their summaries include some of their historically important achievements. We also provide an example of a center that transitioned between institutions (the center focused on aging with a disability). It is also important to note that RERCs are focused not only on research and development of technologies and policies, but also on dissemination of knowledge and training of new researchers. We limit this review to research and development activities, although these other activities are important as well.

To organize this review, one could attempt to group the RERCs (Table 1) by the type of impairment on which they focus (e.g., motor, sensory, cognitive, etc.), by the age of the users they serve (children, adolescents, adults, aging adults), or by their technological focus (e.g. wireless communication, robotics, prosthetics). Ultimately, we chose to group them by the functional need they target, defining three broad categories – mobility, communication and cognition, and rehabilitation therapy and exercise. Most of the RERCs cut across the three categories, but this grouping serves to frame this review into tractable themes.

As a way to gain an overview of the extensive body of work described here, Table 2 provides a timeline of sample product and policy innovations resulting from RERC work. These are products that have come to be used by many people outside of the original RERC research scope. Note that the RERCs have also contributed a large amount of knowledge to rehabilitation besides these practical products and policies. This knowledge is archived in peer-reviewed publications, a sampling of the most important of which are cited in the text of this review. Further, emerging projects continue to aim to generate new products, as described below.

**Table 2:** A sampling of product and policy innovations resulting from the 19 currently active/recently ended RERCs surveyed in this study

1970s
Tactile Vision Substitution Systems for displaying tactile images on the skin [346–348], ultimately leading to devices such as the BrainPort [349, 350].
Sip and puff controls for electric wheelchairs [521]
KEI (Keyboard Emulating Interface) Standard and then commercial KEI’s that enabled assistive technology users to control Apple, IBM, and Linux computers [522]
A three-dimensional database on the anthropometry of wheeled mobility users [200, 523]
“Talking Signs” navigation system for blind pedestrians [358, 359] which spread to many locations around the world and inspired a legion of other related systems
“Sweep VEP” (Visual Evoked Potential) to enable assessment of vision impairments in infants and pre-verbal children [360]
1980s
Some of the first popular devices to help blind people with specific tasks such as liquid level indicators, auditory light probes, an Auditory Oscilloscope, techniques and training materials for electronic circuit design and soldering, Matlab, Computer Numerical Control machines [351–353, 368]
Microprocessor-based talking tactile-haptic educational games for blind children [351]
First set of hardware/software accessibility guidelines for computers were developed by an RERC for the White House Committee on Computer Access in 1985 [522]
Photorefraction methods were perfected for visual screening of young children by merely taking a photograph and having it analyzed [361]
First internal accessibility guidelines used by IBM (1986), the Information Technology Foundation of ADAPSO (ITF) and Microsoft Corporation (who distributed them to all of its developers; used as the starting point for creating their Windows-specific accessibility guidelines) [522]
Three of the first five access features in Apple’s operating system (StickyKeys, MouseKeys, and SlowKeys) were first developed at the Universal interface and information technology access RERC and represented the first access features built into any standard commercial computer operating system. Later, these 3 and 6 additional access features developed at the RERC were licensed (royalty free) by IBM and Microsoft for inclusion in their products. Nine of the first ten access features Microsoft built into Windows 95 (and every version of Windows since) were licensed from the RERC [522]
A robotic fingerspelling hand for deaf-blind communication [357]
1990s
GIDEI (General Input Device Emulating Interface) standard that covered both keyboards and mice [399] and implemented in a commercially adopted hardware device, the Trace Transparent Access Module (TTAM) [400] and a software version built into Microsoft Windows 95 and beyond [401].
The first braille Telecommunications devices for the deaf (TDDs) for deaf-blind users [355]
The first touch-tablet based computer access system for blind users [356]
RERC guidelines were used in creating the first Section 508 guidelines, which contain technical criteria and performance requirements for accessible information technology used by federal agencies [402, 403]
Squirt shape socket fabrication system [44–50]
The Multi-Focal Electroencephalogram system [366] was developed to provide objective assessment of vision function at hundreds of locations on the retina simultaneously. The underlying technology was applied to develop the first brain communication interface for severely disabled individuals with locked-in syndrome [367]
The first web access guidelines were developed by an RERC in 1995 [522]
Chart-based tests (the SKILL card [362], Colenbrander Low Vision Acuity Chart [363], SKRead Test [364], Colenbrander Mixed Contrast Test [365], etc) developed as fast and clinically practical ways of better measuring visual impairment and function
A RERC united 35 different guidelines, to create the Unified Web Accessibility Guidelines, Version 8.0 of which was used as the starting point of the W3C’s Web Content Accessibility Guidelines [408]. The RERC co-chaired and supported both WCAG 1.0 and 2.0 and developed many of the quantification of measures, open-source test tools, and test database for WCAG. Used in US, Canada, Europe, Australia and most other countries
EZ Access keypad and software interface extensions provide access to people with limitations due to vision, hearing, reach, touchscreen use, reading, or cognition [406]. The EZ Access techniques are now implemented in over 50,000 cross-disability accessible kiosks in post offices, airports, museums, memorials etc.
2000-2010
Shape&Roll prosthetic foot [51–64]
Orthotic and Prosthetic Users Survey [88–94]
The training video Keys to Success in SCI Training: Balance and Stability in a Wheelchair [524]
Patient-cooperative training regimes for the Lokomat gait training robot [525]
TMAP, a system to allow blind users to obtain custom tactile maps of any desired area in the US, and a crowd-sourced solution for providing video description [373]
Adaptable prosthetic foot-ankle mechanism [69–73, 141, 172, 526–528]
Development and validation of Impact Damping, Hysteresis, and Loaded Contour Depth test methods for inclusion in the ISO standard of Wheelchair Seating (ISO 16840) [529]
National (RESNA) and International (ISO) standards for design, performance, and labeling of wheelchair transportation safety (WTS) technologies, including WC19 crash-tested wheelchairs for use as seats in motor vehicles [280–286]
Contributed to KineAssist MX, commercialized by HDT Robotics, which uses a force-sensing, pelvic support mechanism to sense the user’s intended walking speed and direction to drive a moving surface, thus allowing a person to move at their own intended speed and pace [454].
Web-based training course, *Evidence-Based Manual Wheelchair Prescription and Practice*, is launched and offered for 6 years [530]
Augmentative and Alternative Communication design features (visual scenes; navigation/ organization/color features) suitable for children and adults, including downloadable web templates [531–535]
A post occupancy building evaluation method for evaluating the achievement of universal design goals [212, 213]
A wireless system which interfaced with public captioning systems to provide captions for recorded and live events on a user’s mobile device. The system was piloted in Redskins stadium in 2009 and used in the Super Bowl at Cowboys Stadium in 2010. The captioning system was licensed to the Monterey Bay Aquarium, University of West Georgia, and Dallas Cowboys [418]
An external alerting interface device enabled people with sensory disabilities to be aware of incoming wireless emergency alert messages. The disability community and Federal government agencies such as DHS, FEMA, FCC, and state emergency management entities [536]endorsements have led to the development of a portable, traveler’sversion.
An arm exoskeleton for upper extremity rehabilitation training after stroke, the ArmeoSpring, sold by Hocoma, now in use in over 700 hospitals and clinics, with subsequent application for rehabilitation for people with spinal cord injury, multiple sclerosis, cerebral palsy, and should injury [450]
Changes to the ICC/ANSI A117 standard, referenced by building codes and used as a source of technical criteria by the ADA Standards, including visitable home design standards and updated standards for wheeled mobility clearances [537]
2010-present
Tiramisu Transit app, a crowd-powered transit information system for smartphones [7, 8]
Created the concept of the Global Public Inclusive Infrastructure (GPII) [416]. Over 50 companies and organizations, and over 100,000 individuals have now joined in the effort. The focus is now on secure necessary funding and moving the GPII from research to real-world implementation and international availability [417].
The App “Factory” concept of rapid development of discrete technology applications that work on contemporary smart devices. Apps for blind/low vision users included Braille readers, currency identifier, and apps for those with cognitive or communication issues including talking photo diaries. Since 2011, eleven mobile apps have been released and have accumulated over 500,000 installations [419]
An open-source middleware framework (called FAAST) to allow interface between markerless tracking technology and freely available games and Internet applications [490]
AIMFREE teleassessment tool (i.e., phone, iPAD, laptop) measures the accessibility of health clubs and fitness facilities (AIMFREE) in real time and is available free of charge to professionals or consumers with disabilities anywhere in the US [538]
The RAPUUD Scale - a product usability evaluation method for assessing universal access [210]
Universal design homes constructed and open to the public in three cities including the LIFEHouse™, two as part of the Wounded Warrior Project, and two in the Horizons Home Show in Buffalo, NY [216]
DOR (Drive-in Occupant Restraint) that improves independent and safe positioning of motor vehicle safety belts [277]
Quantum Securement System, the first fully automatic rear-facing wheelchair securement station [276]
Computer vision technology for solving problems faced by blind people such as reading displays and signs or orienting to a crosswalk [369–372]
ASTM Approved Standards for Universal Design of Fitness Equipment [539]
*SCI HARD* mobile game to enhance self-management skills, health behaviors, and participation among adolescents and young adults with spinal cord injury [384]
Multisensory interactive touch models and maps provide information and orientation assistance to all building users – four installations in educational and rehabilitation settings and 20 in the offices of a major technology company [215].
A wheelchair cushion with adjustable fluid volume is patented and licensed to Ki Mobility [540]
A wheelchair seating system designed for persons who propel with one or both feet is patented and licensed to The Posture Works [541]
Universal Criteria for Reporting the Cognitive Accessibility of Products and Technologies ANSI/RESNA CA-1 [542]
*innovative solutions for Universal Design (isUD™)* provides an interactive platform for browsing innovate solutions for UD, reference designs for designers and design resources that summarize the state of knowledge on a variety of topics related to UD [543]

Note that while some of these RERCs have been funded through multiple cycles stemming back to the 1970s, this table provides only a sampling of the overall RERC output, since 129 RERCs have been funded since 1984

Table 3 shows an analysis of the distribution of disciplines represented in each RERC, in order to allow the reader to assess the combination of expertise that contributed to the technological advances we describe. Note that 70% of the research and development staff of the RERC are engineers, with 23% being researchers in clinical fields, and only 7% being from basic science fields. Thus, while the RERCs certainly are multidisciplinary (a total of 36 fields are represented), they primarily involve engineers. Significantly, about 11% of RERC staff have a disability that gives them personal experience with the problem on which they are working, an ideal that NIDILRR advocates and rewards in its grant review evaluation criteria. Note also that 13 of the 19 RERCs reported interactions with another RERC, consistent in part with a multidisciplinary, collaborative approach.

**Table 3:** Five most common professional fields of the research and development staff of the NIDILRR RERCs analyzed in this paper, shown in engineering/technical, clinical, and basic science categories. Percentage shown is the percent of the 595 total staff reported. Graduate students were included but not undergraduate students. Average number of staff reported per RERC = 35 +/- 13 SD. Also shown are the percentage of these staff who have a disability that gives first-hand experience with the problem on which that staff is working. Other fields besides the top five in each category are also listed; a total of 36 fields are involved in RERC work.

Engineering and Technical Fields	70%	Clinical Fields	23%	Basic Science Fields	7%
Biomed Engineering	19%	Physical Therapy	6%	Social Science	2%
Computer Science	14%	Occupational Therapy	5%	Ecology	0.8%
Mechanical Engineering	9%	Speech/Lang Therapy	4%	Neuroscience	0.6%
Electrical Engineering	7%	Psychology	4%	Exercise Science	0.5%
Industrial/Human Factors Eng	6%	Prosthetics and Orthotics	2%	Health Sciences	0.3%
OTHER:	15%	OTHER:	2%	OTHER:	2.8%
Civil Engineering		MD – PM&R		Biomechanics	
Materials Engineering		MD – Orthoped Surg		Biology	
Information Science		MD – Neurology		Gerontology	
Biostatistics		MD – Other		Cognitive Science	
Urban Planning		Nursing			
Architecture		Pharmacy			
Design		Public Health			
Accident Investigation					
Robotics					
Rehabilitation Engineering					
Has a disability that gives first-hand experience with the problem on which they are working?	5%		3%		3%

## Review

### Centers with a mobility focus

Most of the work at the original Rehabilitation Engineering Centers funded in the 1970’s focused on technologies for individual mobility, including prosthetics, functional electrical stimulation, and control systems for powered wheelchairs. While this line of work continues to advance, producing increasingly better technology, the scope of mobility research has expanded to include technologies and policies that address mobility needs at a societal level, such as mobile applications and universal design, which refers to the process of creating products usable by people with the widest possible range of abilities [4].

#### Accessible public transportation

##### Need and rationale

Many of the known problems with under-employment and social isolation of people with disabilities can be linked to poor transportation within the local community [5, 6]. In most cases, public transit serves as the only reliable option for spontaneous, low-cost, independent travel. Many people with disabilities lack the resources or ability to own and operate their own vehicle and taxi service is frequently unavailable or too expensive. Paratransit serves people who cannot access their mainline service but has problems, like long advance reservations, cost, and poor on time performance. Mainline transit not only supports more independence, but it also acts as an effective vehicle for mainstreaming people with disabilities into the rest of society.

While public transit can provide strong transportation services for many people with disabilities, there are still barriers in most systems. Riders with disabilities frequently encounter challenges in access to information, limitations in boarding and disembarking vehicles, safety risks, and usability problems in the built environment. Best practices are often not followed and there are common problems with regulation compliance.

Information access is a major barrier to using mainline transit systems. For example, early investigation by this RERC team identified only one out of eleven sampled US transit websites that passed accessibility checks with flying colors. The complexity of transit service also puts a premium on time and location dependent, real-time information. Knowing a bus is arriving is of little value if there is no room on the bus to board.

The widespread deployment of low floor vehicles has improved physical access, particularly in buses. Low floors are an excellent example of universal design as the lack of steps also reduces dwell times at stops by speeding up boarding by all riders. However, boarding times by people who use wheelchairs in buses are still slow due to challenges in positioning and securement and the design of fare payment systems.

Even when information and physical barriers are not present, transportation providers are often unaware of best practices in policies and operations. In some cases, individual employees will knowingly violate accessibility policies when other policies have a stronger influence on their performance measurements (e.g., saying “the lift is broken” to stay on schedule).

The scale and complexity of public transit creates significant challenges in detecting problems and, when necessary, funding remedies. Some barriers are caused by poor coordination between agencies, especially where service connects with infrastructure maintained by local municipalities. Difficulties overcoming the *last mile*, or trips between local transit stops and origins or destinations, contributes to dependence on expensive paratransit services.

##### Advances

Since 2008, the RERC on Accessible Public Transportation (RERC-APT) has focused on many of the challenges described above, specifically improving information and physical access through universal design approaches.

**Fig. 1:**
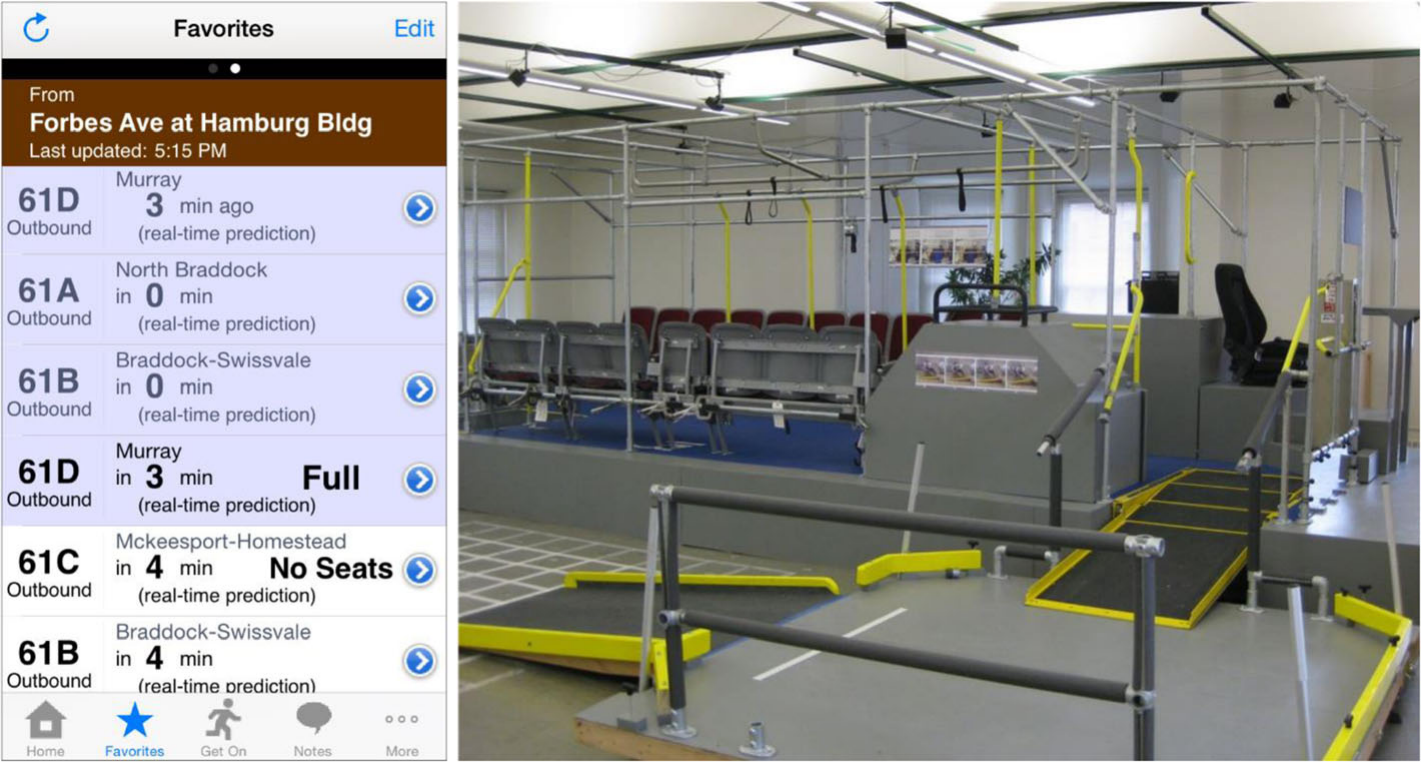
Left: Live screenshot of the Tiramisu app showing real-time crowdsourced arrival and fullness information. Right: The full-scale, reconfigurable simulated bus and the apparatus for testing various ramp slopes

The core manifestation of the information work is the Tiramisu Transit app [7, 8], which is a crowd-powered transit information system for Android and iPhone (Fig. 1). Tiramisu provides easy access to schedule and arrival times and availability of seats, and allows users to share information about problems. This system has been very successful and now acts as a living test bed for a variety of research topics. Tiramisu has received accessibility and industry innovation awards, has almost reached one million user-days, and has collected well over 200,000 crowdsource contributions about real-time transit service. Our universal design approach is also successful; the vast majority of the thousands of users are unaware that their crowdsource contributions are specifically designed to help riders with disabilities.

Tiramisu was developed through a human-centered approach. Early studies by the team revealed key insights on how people want to interact with their transit agencies and fellow riders [9–11], the manner in which people want to report barriers while mobile [7, 12], and how to encourage greater crowdsource contribution from the general public [13]. The team is now measuring the daily, real-world impact of time and location dependent, real-time information on people with disabilities.

For physical barriers, we have focused on issues surrounding rapid boarding and egress from transit vehicles, vehicle ramp slope, and understanding of how local infrastructure impacts the ability to reach transit stops and stations [14–18]. Research has centered on full-scale simulation of transit buses to identify important design elements and new design concepts. These have been incorporated into operational transit buses that were evaluated in the field and proven successful.

Full-scale simulation has also been used to generate critical findings relevant to US Access Board rule-making on vehicle ramp slope. We have also been providing input to regulatory activities related to rail transportation. These activities, combined with close industry collaboration, extend the impact of our findings beyond the academic domain.

Our research on the last mile is utilizing a multi-method approach to identifying barriers and strategies that could enable more people with disabilities to utilize mainline systems. This includes an investigation on how sidewalk quality impacts access to bus stops and stations.

Finally, we are collaborating with the US Department of Transportation to characterize how technology can address barriers and meet the needs of people with disabilities.

##### Future directions

Access to transportation information, especially in real-time, will continue to be an important challenge. Accessible information supports better transportation options awareness, disruption management, spontaneity, and social inclusion. Universal design approaches, like Tiramisu, will create value to all riders and increase support for these information services.

Identification of physical barriers and strategies to avoid them can lead to improvements in efficiency, mostly through faster boarding and egress times. Reduced dwell times will help counteract the perception that people with disabilities slow down bus service. Universal design strategies to simplify securement and fare payment are also needed. Advances in designs for smaller buses will serve users in low-density residential areas in suburban and rural locations. Finally, accessibility in the last mile will continue to impact the utilization of public transit and can reduce dependency on paratransit services.

Unfortunately, service providers and decision makers will continue to make decisions without fully weighing their impact on people with disabilities. Likewise, fellow users of public transportation are often unaware of the needs of people with disabilities. Social computing can create and maintain dialog between all riders and service providers, thereby strengthening the voices of people with disabilities and lead to greater empowerment in transit policy practices at the local level. This should also reinforce the importance of following best practices and help eliminate service-oriented barriers.

#### Manipulation and mobility

##### Need and rationale

**Fig. 2:**
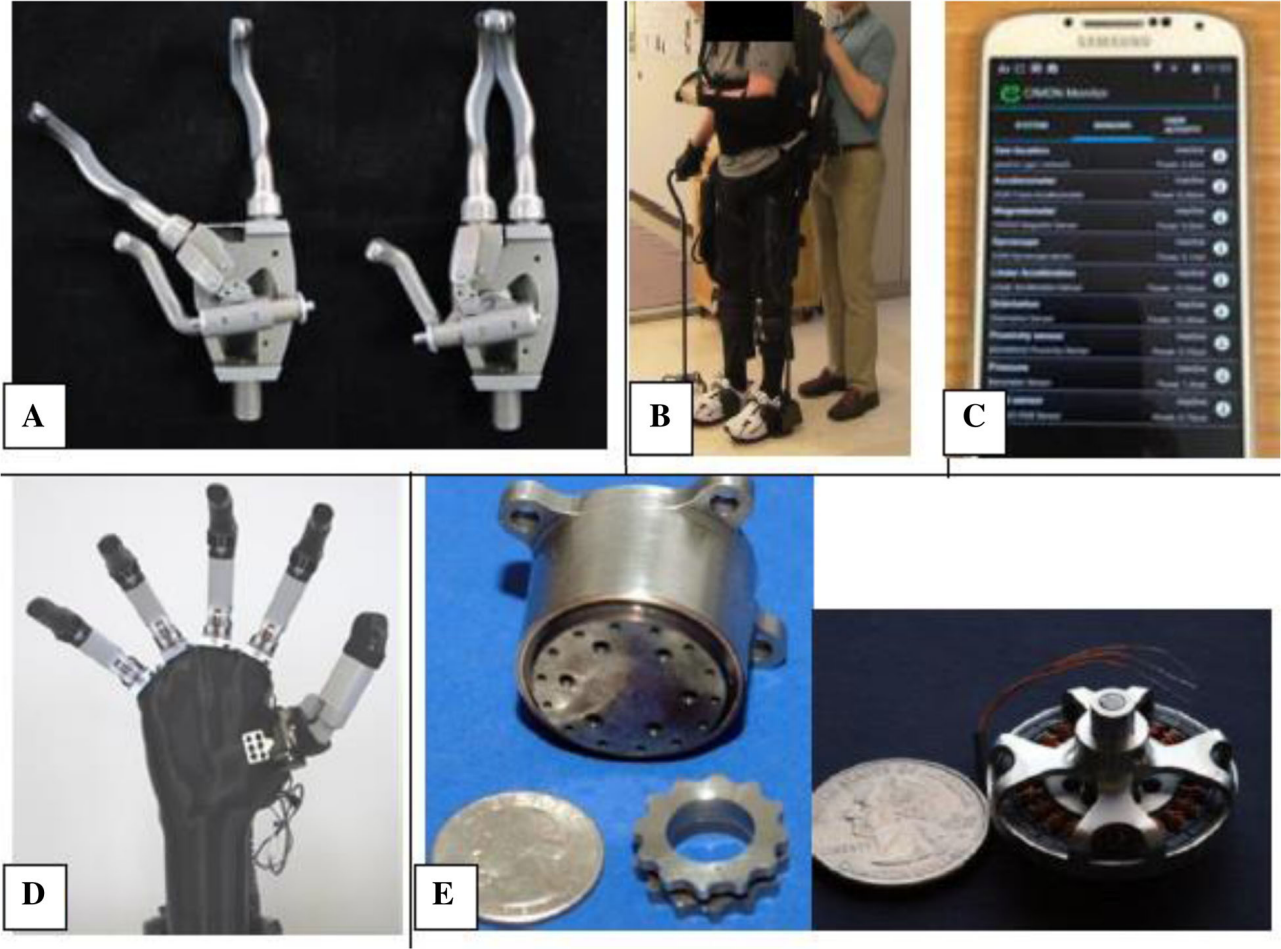
(**a**) Body-powered prehensor in (*left*) voluntary close and (*right*) voluntary open mode; (**b**) Stroke survivor walking with the Ekso (Ekso Bionics).; (**c**) Robust, configurable smartphone interface for monitoring real-life community mobility and social interactions and data analysis; (**d**) Test apparatus for powered ilimb digits (Touch Bionics)—to enable evaluation of control using novel pattern recognition algorithms; (**e**) Small, lightweight cycloid drive transmission (*left*) and exterior rotor motor (*right*) developed for lightweight RIC arm can be leveraged to create a lightweight powered prosthetic leg

Impairments in manipulation and mobility result from a wide range of diseases, injuries, and conditions that cause loss of controlled movement of the arms and/or legs. Interventions may involve devices to replace or augment the function of the impaired limb(s), or therapeutic strategies to improve residual function. The Technologies to Advance Manipulation and Mobility (TEAMM-RERC), established in 2013, comprises six projects that target technological and knowledge gaps within this broad research area (Fig. 2).

Strategies to improve manipulation and mobility were historically built around human-powered devices, such as body-powered upper limb prostheses [19]. With some modifications, these devices are still used today, despite limited function, because they are simple, light, robust, and provide inherent proprioceptive and force feedback [20]. Passive lower limb prostheses provide basic mobility, but, unlike an intact limb, do not provide power during ambulation. Although new materials, such as plastics and carbon fiber have made these devices lighter and able to store energy, walking with a passive prosthesis is still slower, more asymmetric, less stable, and expends more energy than able-bodied walking [21, 22]. This is particularly a problem for the increasing number of older persons with amputations. These smaller, weaker individuals need power for the simple tasks that enable independent living. Despite remarkable achievements—the world record for a 100m sprint for a bilateral lower limb amputee is only one second behind that of an able-bodied runner—passive devices are not suitable for all individuals.

The use of motors to assist human movement began many years ago, but motorized devices still tend to be heavy and costly. Despite the rapid evolution of upper limb myoelectric prostheses since the 1970s, less than 40% of prosthesis users chose them [23] and these costly devices are frequently abandoned—due to weight, limited function, discomfort, and inadequate control systems [24, 25]. Powered, motorized lower limb prostheses offer great promise; however, although computers, controllers, and batteries have become much smaller and more powerful in the last few years, these devices are still too heavy for older individuals. Challenges also remain in providing intuitive control and in ensuring safe, robust performance. The potential of new robotic exoskeletons to improve mobility is huge, but has not yet been clinically realized.

Almost two million Americans use wheelchairs or scooters for mobility; most (90%) use manual wheelchairs rather than the relatively expensive, heavy powered devices [26]. Manual wheelchairs have evolved from heavy, clumsy devices pushed by an attendant to more robust devices powered by the user. New materials, e.g., titanium, have reduced wheelchair weight and enabled design refinement, and we have learned much about improving device deficiencies and wheelchair biomechanics. However, a core problem remains: sitting down for long time periods has physical side-effects—on the digestive, cardiovascular, and renal systems and skin—not to mention the psychological toll of always having to look up at everyone.

##### Advances

This is an exciting time in rehabilitation research, in part because of the ready availability of smart devices with advanced electronics and ever smaller and more accurate sensors that enable more intuitive device control and open up new directions for therapeutic interventions.

The amazing sensor capability built into smartphones is being leveraged within TEAMM to create an innovative new outcome tool that will enable real time, unobtrusive measurement of mobility in homes and community [27]. Latest generation electronics and advanced mathematical algorithms are being used to improve control of motorized multi-function prosthetic fingers—so that people with partial hand amputations can control these advanced devices while independently moving their wrist [28]. Our NIDRR-funded body-powered prehensor—allows both voluntary open or voluntary close capability with the turn of a switch [29]. It is being evaluated in a clinical trial (using onboard sensors) to determine whether people use this functionality in their daily lives.

The first powered leg prosthesis is now commercially available; however, this device is big and heavy. Reducing the size and weight of motors and transmissions remains a significant challenge. Within TEAMM, we are designing a lightweight powered leg that only provides power when really needed – i.e., for activities that are difficult for individuals with transfemoral amputations, such as getting up from a chair or going up stairs and ramps. This focused approach allows us to use much smaller, lighter batteries, motors, and transmission systems.

Exoskeleton systems are being developed as therapeutic tools for various populations with limited mobility. However, the benefits of these complicated, expensive devices for rehabilitation have yet to be determined. TEAMM is evaluating use of an exoskeleton after severe stroke, where severe hemiplegia and other medical issues frequently prevent functional walking. Finally, TEAMM is developing a manually operated wheelchair with a unique ergonomic drive system (similar to a conveyor belt design) that enables the user to be mobile when sitting, standing or anywhere in-between. The chair pulls the user up within the frame using a 4 bar linkage system. Propulsion is done by using a linear track that is connected to the drive wheel with segments of chain. Thus the hand drive has a 1 to 1 movement like using a normal wheelchair wheel, but allows the vertical mobility (and keeps the users hands cleaner).

##### Future directions

Within the next 5 years and beyond we will see both an impressive maturation of some of our current technologies and evolution of new ideas. Powered lower limb prostheses are here to stay and others, including our lightweight design, will become commercially available. Similarly, exoskeletons will be evolved to enhance mobility in specific populations, and will be commercially deployed. Motorized orthotics dedicated to single joint movement, e.g., powered elbow orthotics (like Myomo’s MyPro), powered knee braces, and powered hand assist devices will continue to advance.

Control systems will incorporate improved algorithms and electronics. Surgical techniques—such as Targeted Muscle Reinnervation [30]; direct skeletal attachment of prostheses; limb contouring to enhance the human interface with prostheses and orthoses; and implantable electronic devices to enhance bidirectional communication between brain, spinal cord, nerve and muscle—will enhance users’ ability to benefit from improved control technology. Wheeled mobility will evolve to allow users to negotiate stairs and curbs, or stand while moving: future devices may not look at all like current wheelchairs.

New technologies and materials will continually enhance existing devices. However, a deep clinical understanding of user needs is essential to advance technologies for people with disabilities. As in all rehabilitation research, understanding the marketplace and obtaining input from end users and clinicians during early development is essential to ensure clinically and commercially viable interventions.

#### Prosthetics and orthotics

##### Need and Rationale

The field of prosthetics and orthotics (P&O) deals with the provision of assistive devices to persons with physical disabilities, often movement disabilities. As a profession it exists at the intersection between engineering and health care, dealing intricately with the interface between the person and technology. Since 1972 when Department of Education funding for the P&O RERC began, interventions based solely on passive mechanical devices have been replaced by microprocessor controlled devices, some with implanted control systems. Research is needed to effectively and economically translate this level of functional restoration to the broader population of P&O users and provide evidence to support intervention effectiveness.

##### Advances

Since the inception of the RERC program in 1972, the integrated education and research missions of the P&O program at Northwestern University (NU) have provided a unique environment for the NU-RERC. In this setting, engineering graduate students have interacted directly with clinical faculty and students being clinically trained to provide P&O services to individuals with disability. The proximity of the NU-RERC to the Rehabilitation Institute of Chicago (RIC) and Northwestern Medical Campus has also meant that device users can interact with researchers. Synthesis of interactions among stakeholders facilitated by this environment and funding from NIDRR is what has allowed the Northwestern University Prosthetics-Orthotics Center (NUPOC) to remain a leader in P&O research and education.

Dudley Childress, Ph.D., began work at Northwestern in 1966, leading development of myoelectric control systems for the DC motors needed to drive artificial hands and arms and new self-contained and self-suspending socket designs [31–41]. This early prosthetics research was soon noticed by the newly formed National Institute on Handicapped Research (NIHR) under the Department of Health, Education and Welfare, which selected Northwestern to become one of five new RERCs. Led by Dr. Childress, the initial NU-RERC had two focus areas: (1) assistive equipment for persons with disability, and (2) total knee joint replacements. The most recent NU-RERC cycle (2008-2014) led by Stefania Fatone, Ph.D., and Steven Gard, Ph.D., comprised 12 projects focused on clinically-relevant problems in P&O intended to support evidence-based practice, a need clearly articulated in the NU-RERC for P&O State of the Science Meetings in 2006 [42] and 2012 [43].

**Fig. 3:**
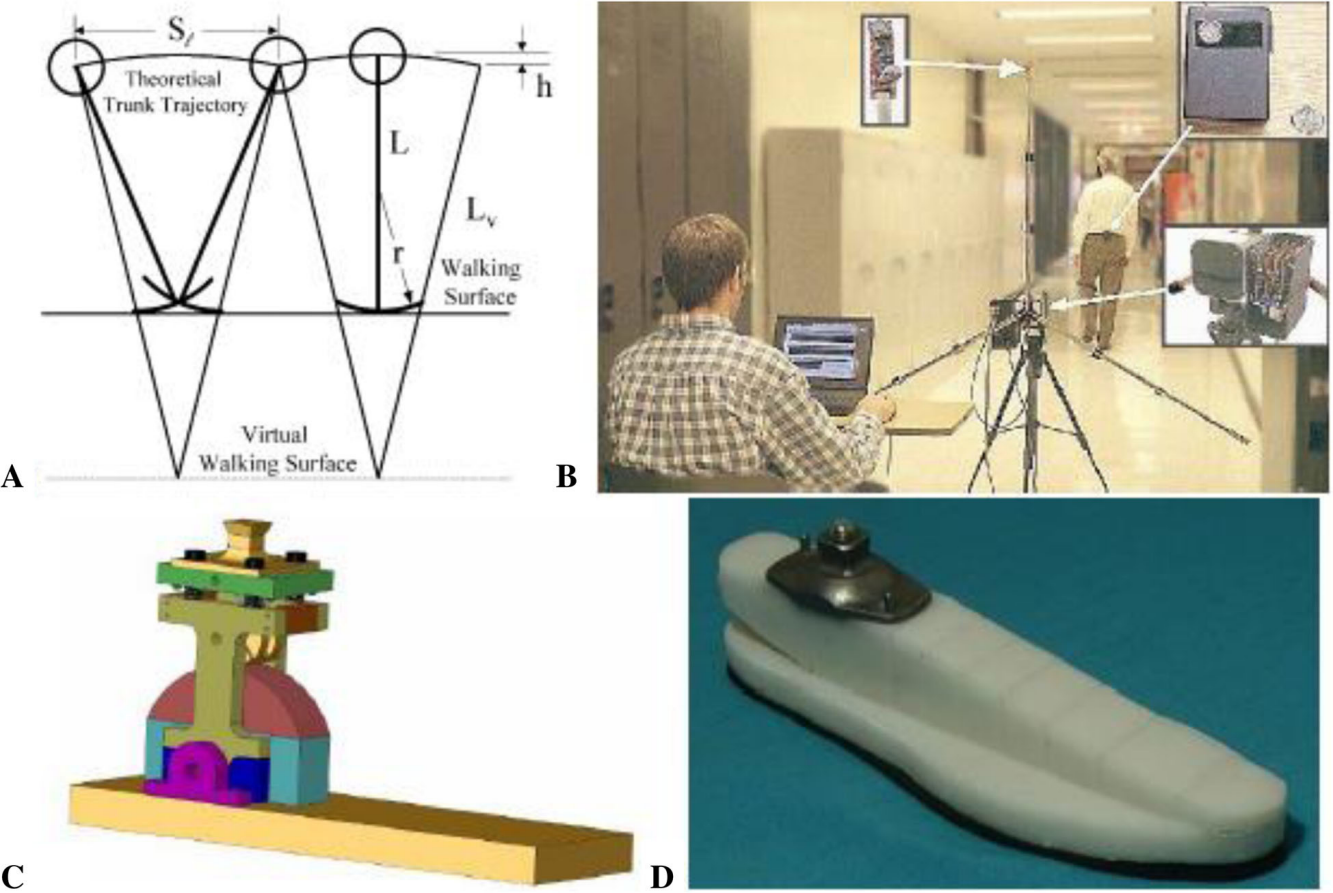
In relatively recent years, NU-RERC for P&O projects have involved the development of: **a**) a rocker-based inverted pendulum model for increasing understanding about human gait; **b**) the Direct Ultrasonic Ranging System (DURS) for acquiring simple gait measures; **c**) a prosthetic foot-ankle mechanism that would adapt to slopes; and **d**) the Shape&Roll Foot, a low-cost easy-to-manufacture foot for use in developing countries.

During the three decades of NIDRR funding many special tools and devices, now routinely and widely used, were developed by the NU-RERC’s engineering staff and students for persons with disabilities (e.g., sip & puff controls for electric wheelchairs, accessible communication systems, environmental controls, squirt shape socket fabrication system [44–50], Shape&Roll foot [51–64], adaptable prosthetic foot-ankle mechanism [65–73], direct ultrasonic ranging system [74–87], the Orthotic and Prosthetic Users Survey [88–94], etc.) as well as important concepts (e.g., extended physiological proprioception [95–102], early exploration of socket interfaces [103–128], inverted rocker based pendulum model for bipedal walking [129–132], challenging the six determinants of gait [133–136], roll-over shape [61, 63, 67, 137–184], etc.) (Fig. 3).

##### Future directions

The field of P&O abounds with well-respected clinical expertise, but is severely lacking in objective scientific evidence to support clinical decision-making. Much of the evidence that practitioners rely on for making decisions about component selection, fitting and fabrication of prostheses and orthoses is anecdotal and undocumented. There has been widespread and growing recognition by the P&O profession of the tremendous need for more research in the field due to the paucity of data regarding patient outcomes that are increasingly being scrutinized. More than ever, clinicians are expected to support their decisions regarding P&O interventions utilizing evidence-based practice. Studies of current P&O interventions that evaluate the effectiveness, elucidate the mechanism of action, and determine the impact on users lives are desperately needed to create the evidence to support practice. The NIDRR-RERC for P&O provided an ideal mechanism for facilitating communication and collaboration between prosthetists, orthotists and engineering researchers with the ultimate goal of addressing clinically-relevant research and development problems and providing improved quality of life to prosthesis and orthosis users.

#### Technologies for children with orthopedic disabilities

##### Needs and rationale

**Fig. 4:**
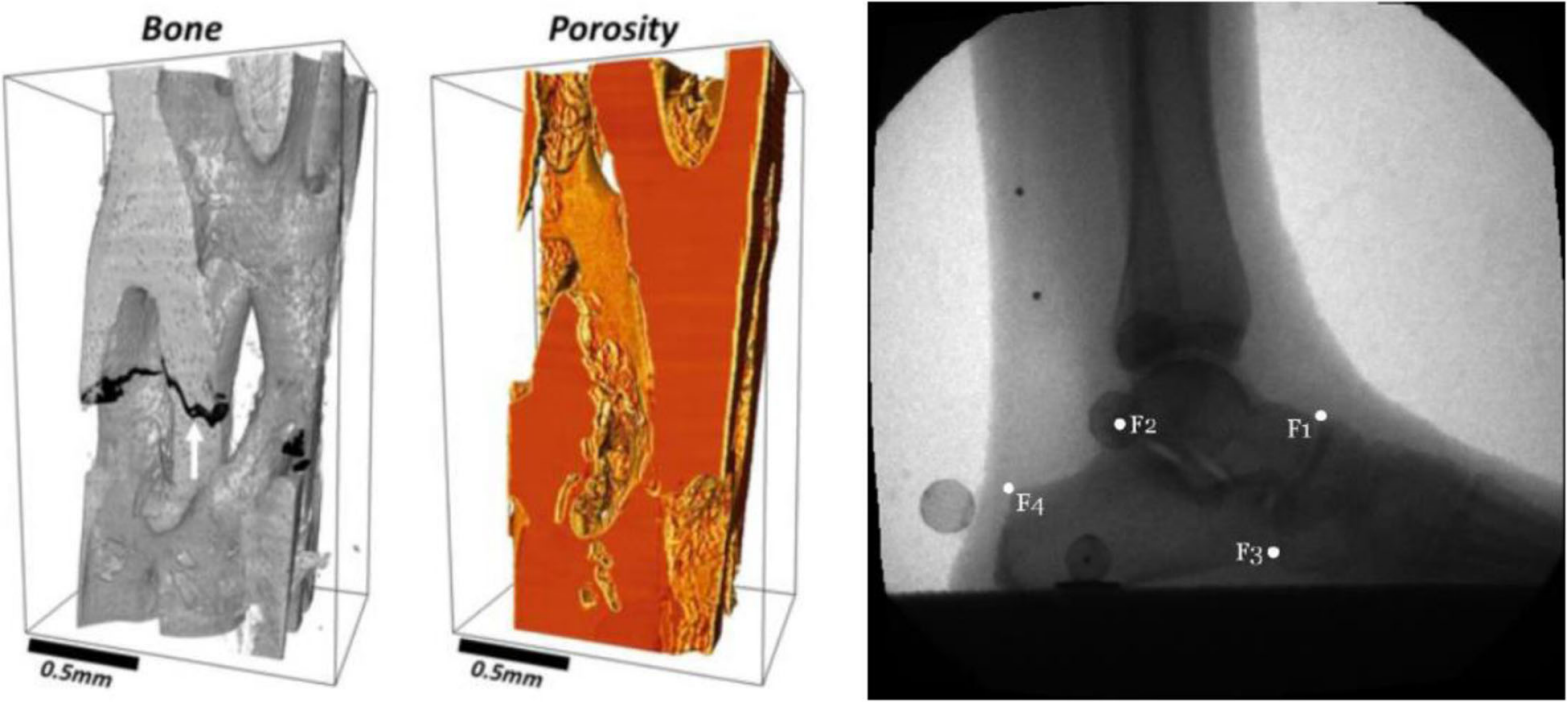
Left: Depiction of cortical porosity in Osteogenesis Imperfecta bone using synchrotron micro-computed tomography. ALS, Lawrence Berkeley National Laboratory. Right: Midstance hindfoot image during normal ambulation from a fluoroscopy gantry (90-110 kVp, 0.5-1.7 mA, 120 sps). Anatomic landmarks on the talus and calcaneus are denoted F_1_ – F_4_.

The four focus areas of this RERC are tissue mechanics, imaging, pediatric robotics, and mobility and manipulation (Fig. 4). In the area of tissue mechanics, very little was understood about the mechanical properties of bone in children with Osteogenesis Imperfecta, an inherited disorder of collagen synthesis resulting in a lifelong risk of increased fractures. With regard to clubfoot, a congenital deformity affecting children and presenting with bone and soft tissue malformation, little was known about the mechanical performance of casting materials used to treat clubfoot, or about the underlying medial fibrotic mass tissues associated with the orthopedic deformity. In the imaging area, virtually no information was available regarding neural tractography or the potential relationship between structural connectivity and functional impairment in the brains of children with cerebral palsy. Motion analysis of the pediatric foot, while possible through optical tracking and traditional marker-based gait analysis, was prone to error due to limited accuracy of marker placement and skin motion artifact. Data on *in vivo* bony motion of the hind foot during gait were simply not available. In the area of pediatric robotics, conventional approaches to spasticity reduction sought either to block abnormal neural activity through botulinum toxin or baclofen injections or to adjust muscle fiber length through stretching, orthoses and serial casting. There was no effective and convenient robotic approach to incorporate combined voluntary movement and passive stretching to reduce impairment and improve function. Another robotic challenge was the lack of an effective approach to treat lower limb deformity in the axial and frontal planes and the resulting loss of axial/lateral control and stability. Available locomotor training systems were also limited. Few degrees of freedom were available for treatment; little capability existed for passive training; and little was available to motivate participation. Finally, in the mobility and manipulation area, there were few multi-segmental studies of planovalgus foot deformity in children with cerebral palsy In addition, the motion models necessary to address important upper extremity challenges in children with orthopedic disabilities had not been developed. Evidence supporting the long-term efficacy of technologies to improve manipulation in children were sparse and essentially limited to the adult stroke population.

##### Advances

Work to date on Osteogenesis Imperfecta bone through microstructural analysis has revealed abnormally elevated vascular porosity in these children within regions normally occupied by dense cortical bone. Novel mechanical testing has provided measurements of bone material properties at the macroscopic level including modulus, strength and findings of anisotropy. Reduced bone strength in these children is largely attributed to elevated cortical porosity [185]. Kinematic analysis of cast materials during simulated clubfoot treatment shows minimal creep for plaster of Paris and two synthetic cast materials. The synthetic materials are more rigid during the early stages of casting, resulting in reduced overall creep [186]. Mechanical analysis of MFMT tissues indicate unique specimen characteristics which may be related to the tissue ultrastructure. Further histological analysis has been effective in identifying specific tissue fiber characteristics and the distribution of tissue constituents within each clubfoot specimen.

Brain structural imaging now provides high angular resolution diffusion imaging (HARDI) in which data are used to construct higher order models of diffusivity. These models are used for diffusion and probabilistic tractography, in which tracts are modeled based on likelihood of structural connectivity in three dimensional space. The RERC has helped to develop voxel-based approaches with metrics of overall brain connectivity to predict functional impairments [187]. A biplane fluoroscopy system has been developed for noninvasive, 3-D foot and ankle motion analysis. The system supports biplane (3-D) fluoroscopy and ground reaction force measurements for kinematic and kinetic analysis. Validation of the imaging system combined with markerless tracking software has been done with the system being employed for several clinical applications in children and young adults [188].

A combined robotic strategy has been implemented that provides voluntary movement training and passive stretching of the lower extremities in children with CP during both lab-based and in-home training [189]. The therapy utilizes a portable ankle rehabilitation robot. Significant improvements are being seen most notably in the home-based group and include dorsi- and plantar-flexor strength, passive and active ranges of motion. To treat lower limb deformity in the axial and frontal planes in children with CP, a novel off-axis elliptical trainer has been developed [190]. Children with CP are showing significant improvements with reduced pivoting instability, improved isometric strength, increased balance, and decreased toe-in angle during gait. Subjects with patellofemoral pain are showing improved knee function, proprioceptive acuity, and neuromuscular control. Work in robotic locomotor training has resulted in a cable-driven system that increases active involvement [191]. The new device is more effective than standard assistance training in improving locomotor function and offering pelvic assistance to improve over ground walking in children with cerebral palsy.

A multi-segmental foot model with radiographic indexing was applied to evaluate kinematics in children with cerebral palsy who presented with rigid planovalgus foot deformity [192]. Results show decreased forefoot plantar flexion and increased abduction, and decreased ranges of motion during push-off. Advanced computational models of the UE have been developed that compute 3-D motions and forces at the shoulder, elbow and wrist during movement with wheelchairs and assistive devices (walkers, and crutches). Interestingly, we have found that complaints of pain are minimal in children, despite the orthopedic disability. We believe this may be related to another finding that children employ a variety of mobility and loading patterns including ‘unclassified’ patterns, which are very different than those used by adults. Clinical intervention has been recommended as well as modifications of existing guidelines to better accommodate growing, skeletally immature children [193].

##### Future directions

A key question for Osteogenesis Imperfecta bone tissue characterization is how to accurately assess bone fragility *in vivo*? What are the relationships between genotype and bone properties? How do mechanical properties correlate to image metrics. There are several questions regarding clubfoot tissue that will be key to future progress. What are the relationships between genotype and MFMT properties in those with resistant and recurrent clubfoot? How do we assess MFMT mechanics *in vivo*? What are the direct implications of MFMT mechanics to conservative treatment duration and recurrence of the deformity? How do MFMT mechanics affect the longer term stages of treatment (bracing)?

Future work in imaging will continue to address the question of how to integrate functional connectivity and structural information with a focus on voxel-based approaches. The goal is to provide objective measures of connectivity that can predict functional outcomes, specifically in children with cerebral palsy. In fluoroscopy the question now is how to best deploy the technology. What is the optimal dynamic correction of the pediatric foot with fixed planovalgus deformity? What orthotics and footwear are most appropriate for dynamic hindfoot correction and balance?

In pediatric robotics, there are opportunities for improvement of pediatric ankle therapy through the use of portable in-home approaches. There is a need to adjust treatment parameters dynamically to ensure the effectiveness of home-based therapy. There are opportunities in elliptical training to improve treatment of lower limb deformity in the axial and frontal planes of children with cerebral palsy. With regard to cable-driven locomotor training there is an opportunity to develop intention-driven robotic gait training. Transcranial direct current stimulation is a promising noninvasive technique for modulating cortical excitability which may be more effective in improving locomotor function in children with cerebral palsy. Development of robotic systems including hippotherapy for improving dynamic balance in children with cerebral palsy represents yet another future opportunity.

Finally, in the mobility and manipulation area*,* analysis of triaxial, multi-segmental foot data during gait and other activities provides an opportunity to improve pre-treatment planning and post-treatment follow up. Quantitative upper extremity modeling will continue to increase our understanding of the linkages among pediatric wheelchair propulsion patterns, joint biomechanics, pain, and quality of life.

#### Universal design and the built environment

##### Need and rationale

Over the last 40+ years, a great deal of effort has been devoted to making the built environment accessible. Accessibility laws like the Architectural Barriers Act (1968), Section 504 of The Rehabilitation Act of 1973, the Fair Housing Act Amendments (1988), and the Americans with Disabilities Act (1990) specify minimum requirements to ensure that the built environment does not discriminate against people with disabilities. Experience with accessibility laws led experts to recognize the need for a different approach to design of the built environment, which Ron Mace and Ruth Lusher termed “universal design” [194–196]. The premise for this new approach was that the environment can be much more accessible than laws can realistically mandate on the basis of non-discrimination. If more attention were given to improving function for a broad range of people, they argued, a usable world for people with disabilities would become the norm.

When the RERC on Universal Design and the Built Environment (RERC-UD) was first awarded in 1999, many barriers to universal design’s full integration and implementation existed. First, there was a need to clarify and improve the definition of universal design (UD) and the well-known Principles of Universal Design. Second, there was a need to address critical gaps in the knowledge base. Third, there was a need to demonstrate how to implement UD. Fourth, there was a need to develop mechanisms through which UD could be implemented in practical forms. Fifth, there was a need to address new target populations to expand the community of practice in UD and, in particular, support key change agents to diffuse the concept within their stakeholder groups.

##### Advances

The mission of the RERC on Universal Design and the Built Environment (RERC-UD) has been, and continues to be focused on the advancement of universal design. Over three cycles of NIDRR funding, the RERC-UD has evolved from focusing on justifying the need for UD, to providing evidence to support UD, to evaluating the implementation of UD. Thus, the RERC-UD has made significant progress addressing the existing needs. To help clarify the concept of universal design, the RERC-UD created a new definition of UD that addressed problems identified by critics: “Universal design is a process that enables and empowers a diverse population by improving human performance, health and wellness, and social participation” [197]. The revised definition was then supported by the eight Goals of Universal Design: Body Fit; Comfort; Awareness; Understanding; Wellness; Social Integration; Personalization; Cultural Appropriateness [197]. These goals recognize disability prevention and social participation as important outcomes and also address criticism that universal design is only applicable to a high income context.

Research achievements addressed critical gaps in the knowledge base and provided evidence to support the need for universal design. In its first cycle of funding, the RERC-UD developed the only three-dimensional database on the anthropometry of wheeled mobility users and demonstrated that accessibility standards needed to be revised to reflect contemporary wheeled mobility realities (see, for example [198–201]).

**Fig. 5:**
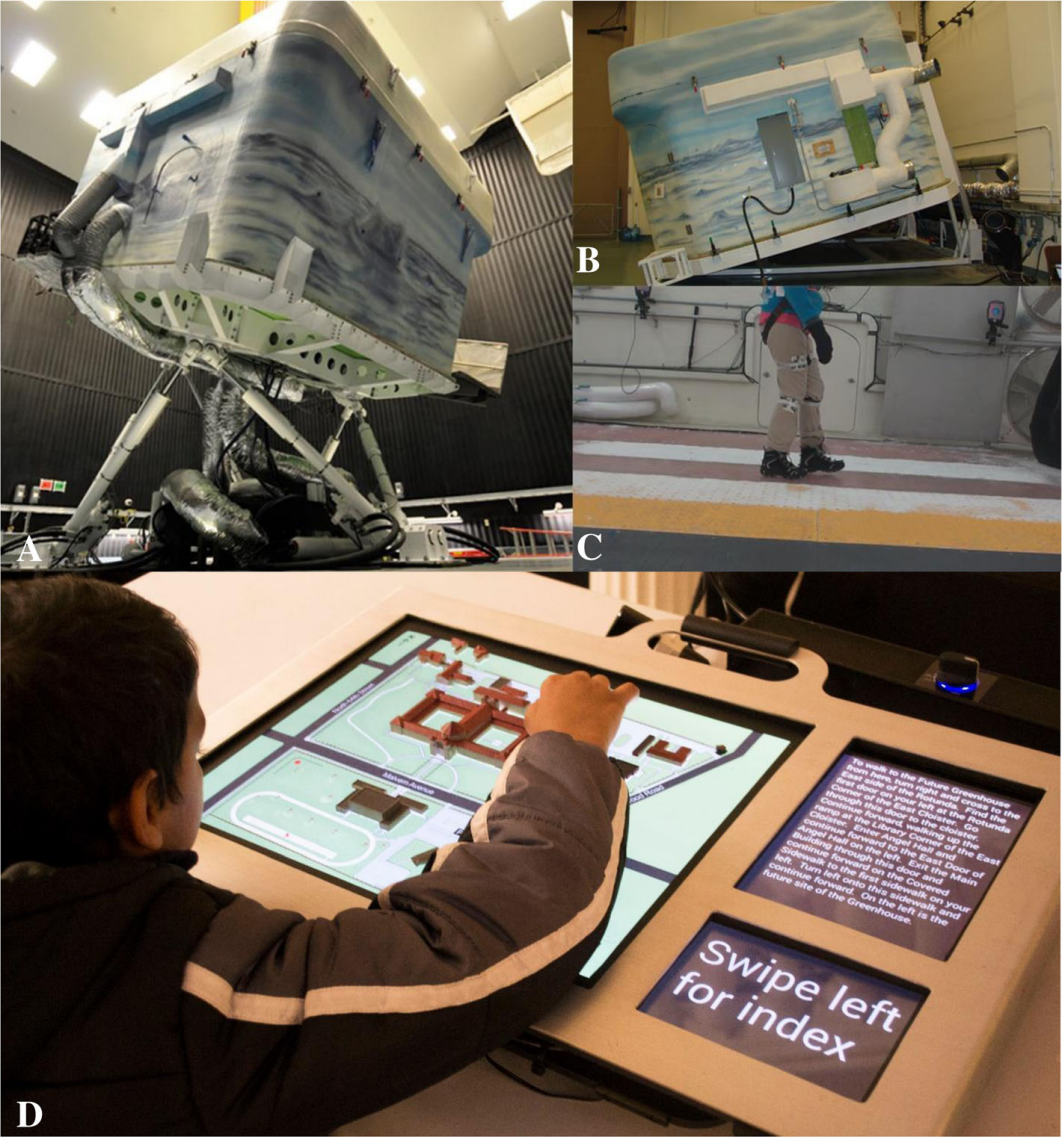
Using simulated environments and interactive technologies in Universal Design research (**a**) WinterLab (one of the Challenging Environment Assessment Labs) on the motion base to create different terrains. (**b**) WinterLab on the Single Axis Base to create slopes. (**c**) A participant walking inside WinterLab. Based on our survey study [544], we found that the key elements decreasing winter accessibility were icy sidewalks and puddles at street crossings and curb ramps. The Americans with Disabilities Act Accessibility Guidelines (ADAAG) allow a maximum run of 9 m (30 ft) for slopes between 1:12 and 1:16. Our study [208] demonstrated that ice covered slopes with a grade of 1:12 were not acceptable for long ramps (>=4 m) even among able-bodied older adults. (**d**) A multisensory interactive touch model installed at the Overbrook School for the Blind that aids in wayfinding [215].

Additional funding led to collaborations with an advanced simulation laboratory Challenging Environment Assessment Lab (CEAL, Fig. 5a-c), and human factors research on stairways and sidewalks in cold weather climates, to demonstrate the value of using simulated environments in UD research [202], (see, for example [203, 204]). CEAL is the world’s first hydraulic motion simulator that can mimic everyday environmental challenges faced by older people and those with disabling injury or illness. Using a multitude of customizable testing environments, CEAL is able to recreate conditions such as ice and snow, different terrains and slopes. Winter presents many challenges to the safety and mobility of vulnerable older people and people with disabilities. The number of falls in winter conditions has been increasing [205, 206]. This has created a sense of fear and discomfort for people to leave their homes [207], and limited their independence by socially isolating themselves indoors. Research [208] demonstrated that ice covered slopes with a grade of 1:12 were not acceptable for long ramps (>=4 m) even among able-bodied older adults. This implies that any exposed sloped surface like a building entry ramp should be cleared diligently, heated or under cover. Findings helped develop a new footwear test method for the ASTM footwear committee and plans are underway to develop a meaningful and easy to understand labeling system for winter footwear [209], as well as new technologies to increase the slip-resistance of footwear. These research findings also support the adoption of universal design strategies like covered or heated ramps and approaches to buildings.

Subsequent cycles of funding have also supported research initiatives on home modifications and rights-of-way. Research activities also led to the development of a suite of tools for evaluating UD products and environments: 1) a usability testing method that introduces UD as an outcome; 2) a method for assessing priorities of end users of products and environments [210, 211]; and 3) a post occupancy building evaluation method for evaluating the achievement of UD goals [212, 213].

To demonstrate how to implement UD, the RERC-UD developed an industry partnership program to advance the adoption of universal design by providing technical assistance in the product development process. Over 20 products and buildings have been completed or are in construction to date including the highly successful and replicated multisensory wayfinding model [214, 215] (Fig. 5d). The RERC-UD has designed and, with builders, built eight UD homes in three cities including the LIFEHouse™, two as part of the Wounded Warrior Project, and two in the Horizons Home Show in Buffalo, NY. The LIFEHouse™ has won several awards for its design, including a national award from the National Association of Home Builders [216].

In an effort to engage in mechanisms through which UD could be implemented in practical forms, the RERC-UD regularly participates in standards development activities. During a previous funding cycle, the RERC-UD advanced the expansion of the U.S. visitability movement by writing a comprehensive policy brief for the AARP Public Policy Institute, initiating and helping the ICC/ANSI A117 standards committee adopt a consensus standard on visitable housing, and providing technical assistance that resulted in the construction of thousands of visitable homes. The RERC-UD also translated the findings of its anthropometry project to implement key changes to the ICC/ANSI A117 standard, referenced by building codes and used as a source of technical criteria by the ADA Standards. The changes will provide larger clearances to accommodate contemporary wheeled mobility users. Most recently, the RERC-UD co-founded the Global Universal Design Commission and developed the first consensus standards on UD. To further formalize and document the implementation of UD, the RERC-UD designed and then obtained approval from the U.S. Office of Patents and Trademarks for a UD certification mark that can now be used in certification efforts.

Key books include the second edition of the Universal Design Handbook, 2E [217], the first comprehensive textbook on Universal Design, Universal Design: Creating Inclusive Environments [197], and a tool for housing designers, Inclusive Housing: A Pattern Book [218].

##### Future directions

Despite numerous successes, additional efforts are needed to further advance universal design and make it a mainstream practice. Over the next five to ten years, the key rehabilitation engineering questions include the following. How can knowledge translation from rehabilitation science be applied to advance standards in both UD and accessibility regulations? What kind of evidence can be gathered to demonstrate the business case for adoption of UD in the private sector and the public sector? How can we provide potential adopters with a concrete means to demonstrate achievement of UD outcomes that has value to them? How can collaborations with related movements advance the adoption of UD, e.g. age friendly communities, housing for aging in place, complete streets, design for healthy living, sustainable design, etc.? How can a critical mass of advanced students be recruited to expand research and practice capacity in the field of UD?

#### Wheeled mobility and seating

##### Needs and rationale

In the US, over 3.3 million persons over 15 years of age use a wheelchair [219]. This is a widely disparate group that varies across many medical conditions and functional presentations. The rehabilitation engineering program at NIDRR had a longstanding focus on wheeled mobility and seating, supporting dedicated centers between 1976 and 2013. Wheeled mobility changed drastically over those three decades. There are now a wide range of commercially-available wheeled mobility and seating devices ranging from fairly simple to highly complex. However, the existence of a range of technologies has not yet translated to improving the health, activity and participation of wheelchair users. Over one-half of users pay for their own devices, greatly limiting access to needed technology [26]. Moreover, less than 25% of wheelchair users are employed, clearly an unacceptable outcome. These statistics reflect an opportunity for research to impact public policy and clinical practice.

The challenge lies in applying science and engineering to answer complex questions and tackle complex needs that are clinically-relevant. This challenge is complicated by the fact that wheelchairs and seating systems are not purely medical devices. In fact, their role as functional devices is probably paramount for wheelchair users. Therefore, research and development activities must generalize to real-world use in order to be clinically-relevant.

##### Advances

The most recent RERC on wheeled mobility (mobilityRERC) operated out of the Georgia Institute of Technology in collaboration with Duke University, Shepherd Center and Georgia State University. It was initially awarded in 2003 and re-award in 2008. The overall focus of the mobilityRERC concerned the use of wheeled mobility and seating in everyday life.

This focus included multiple projects seeking to understand how people obtain and use mobility devices (Table 3). We developed the capacity to monitor the use of equipment during everyday use. This line of research studied the use of power wheelchairs [220], power tilt-in-space seating [221–223], and manual wheelchairs [224]. Consistent across all these technologies was the finding that full-time wheelchair users spend about 12 hours per day in their wheelchairs. This clearly underscores that wheelchairs are used as more than a means of conveyance, rather, wheelchairs are a functional extension of the users. Understanding wheelchair use also focused on persons who sometimes ambulate and other times require wheeled mobility [225–228]. This is an under-studied group who, in fact, represent the largest cohort of wheelchair users. Our recent studies provided evidence that part-time users are uniquely positioned to assess current and anticipated mobility needs [226] and involvement of a trained clinician leads to better outcomes [228].

NIDRR’s RERC program also has a long history of successful design and development projects. Given the changes in the industry, design, and development activities focus on two areas, orphan technologies, and standards development. Test methods and standards are used by manufacturers, policy-makers, clinicians and users to characterize device safety, performance and durability. RERCs have been long-standing members of both national (ANSI-RESNA) and international (ISO) standards granting bodies and integral to wheelchair and wheelchair cushion test development. The mobilityRERC has recently focused on validating test methods on wheelchair cushion impact dampening [229], and interface pressure and has designed a new compliant instrumented buttock model to measure cushion performance [230]. The mobilityRERC is also focusing on valid measurement of wheelchair propulsion torque. We developed a robotic system capable of measuring the forces required to propel manual wheelchairs during over ground maneuvers that include starts, stops, and turns [231]. This novel approach informs both the design and clinical prescription of wheelchairs. Nearly every configuration decision impacts inertia and/or friction of the wheelchair- the two principals that govern propulsion effort. Our approach represents the first opportunity to measure these influences on a systems level to assess how frame type, weight distribution, caster size, drive wheel design, and tire type influence propulsion torque. This effort has already disseminated clinically-relevant information to clinicians and users via non-research based avenues [232, 233].

The seating and mobility industry has evolved to be dominated by a few very large companies. Market forces often prevent these large companies to develop orphan or niche technologies that serve a limited number of people. This development remains the focus of small companies and inventors who identify needs and innovate solutions. The mobilityRERC supported this community by assisting 47 small companies over a 5 year period in a variety of manners. The process started with a presentation of the device by its inventor which was attended by mobilityRERC engineers, designers, and clinicians. This collection of staff brought wide-ranging expertise to device evaluation with respect to function, technical operation, usability, and policy implications. Most inventors do not have expertise in all these areas, so the RERC review was able to fill a void in their knowledge base. After a report was sent to the inventors, the RERC engaged them to determine if they had further needs requiring our assistance. Some products that went through this process are now under production or pre-production and include Rowheels (Rowheels, Inc), Suspension Seat (The Posture Works), Kinetic Innovative Seating System (Kinetic Innovative Seating System, LLC ); Webseat (Tamarak Habilitation Technologies)., X-fer Rail (now sold by Maddak), Sil-Air foam (now sold by Pride Mobility and The Posture Works) and the Stand-up Walker (now with Edison Nation Medical).

##### Future directions

The mobilityRERC has led two State of the Science conferences that gathered researchers, clinicians, and users to discuss current knowledge, and more importantly, the needs of the wheeled mobility and seating community [234–245]. Not surprisingly, stakeholders view technology as a means to access educational, vocational, and leisure activities in addition to meeting medical needs. There is still a paucity of clinically-relevant and valid information about wheelchair and seating system performance that can inform clinicians and users, as well as payers. This lack of information is resulting in restricted access to technology and stifles technology innovation. Because of the overlying functional nature of wheeled mobility and seating, traditional medical research methodologies do not apply. It is time to apply rigorous scientific and engineering approaches to 1) document the outcomes of wheeled mobility and seating, and 2) characterize device performance in valid and clinically-relevant manners, and 3) support innovation of new devices that can be made available to users.

#### Wheelchair transportation safety

##### Need and rationale

Following establishment of the National Highway Traffic Safety Administration (NHTSA) in the mid-1960s, major improvements have been made in transportation safety for people who use seats and restraint systems provided by vehicle manufacturers that are regulated by federal motor-vehicle safety standards. During this same time, increasing numbers of people with physical and/or cognitive disabilities have been traveling in motor vehicles seated in wheelchairs due to legislation that has made motor-vehicle transportation more available and accessible to this population of travelers but that has done very little to address the safety and crash protection for these individuals [246–249].

Recognizing the lack of a reasonable level of transportation safety for travelers seated in wheelchairs due to the use of aftermarket unregulated and often improperly installed and/or used belt restraint systems, as well as seats (i.e., wheelchairs) that were not designed for use in motor vehicles, research and testing was conducted from the late 1970s through 2000 as limited funding allowed. Much of this work was performed at the University of Michigan Transportation Research Institute (UMTRI), and researchers from UMTRI and the Wheeled Mobility RERC at the University of Pittsburgh simultaneously led the development of national and international wheelchair transportation safety (WTS) standards to address the design and performance of wheelchair tiedown and occupant restraint systems (WTORS) and wheelchairs used as seats in motor vehicles [250, 251].

In 2001, the NIDRR announced a priority for an RERC on Wheelchair Transportation Safety (RERCWTS) and two successive five-year grants were funded. The justification was based upon several needs and rationale. ADA regulations do not adequately address transportation safety and crash protection, especially with respect to wheelchairs used as vehicle seats. The provisions of the initial WTS standards and practices were based on very fundamental principles of occupant protection in frontal motor-vehicle crashes for able-bodied passengers and did not address the nature and specific causes of injuries to occupants seated in wheelchairs. The original standards also established performance requirements based on nominal “worst-case” frontal crashes of private vehicles, and did not provide for different approaches to wheelchair securement and occupant restraint that are more compatible with lower crash environments of public transportation systems. Significant usability and accessibility issues also existed with ingress/egress of occupants in wheelchairs, as well as with wheelchair securement and occupant restraint. In addition, the original WTS standards did not address the common practice of adding aftermarket and customized seating systems and peripheral equipment to wheelchairs.

##### Advances

**Fig. 6:**
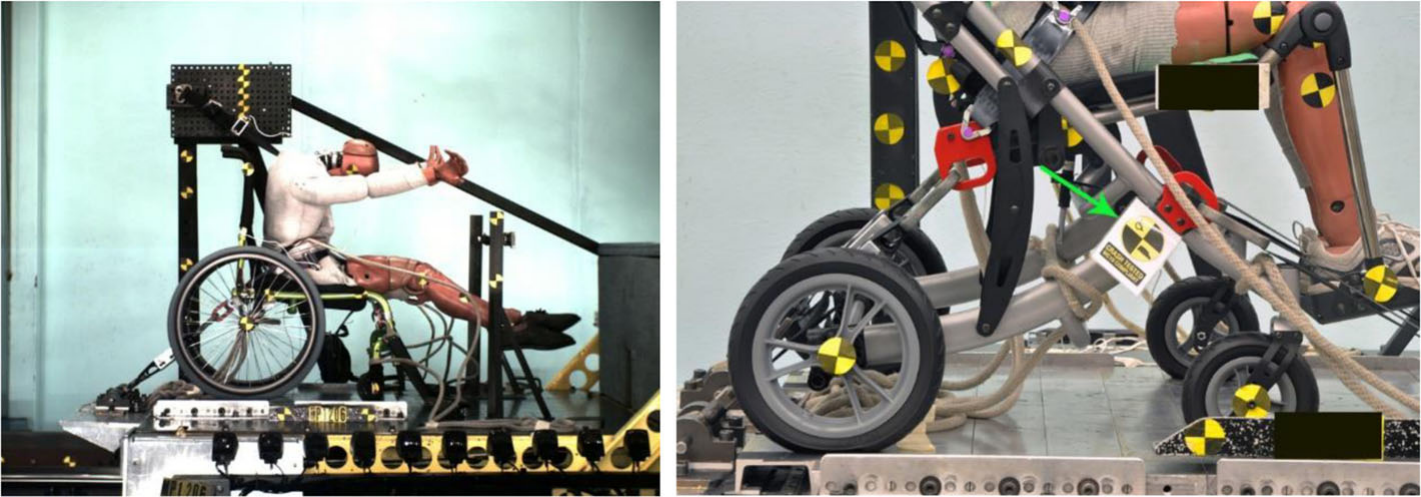
Left: Peak-of-action photo during WC19 frontal-impact sled test of wheelchair with four-point, strap-type securement points. Right: WC19-compliant wheelchair with RESNA Volume-4 logo on permanent label indicated by green arrow

Since 2001, significant and important progress was made on providing the appropriate balance of transportation safety, usability, and independence for travelers seated in wheelchairs in all types of motor vehicles and modes of transportation, including private vehicles, school buses, and paratransit/public transit vehicles (Fig. 6). The primary RERCWTS goals to achieve this were: to (1) understand and describe the issues and injury risks associated with WTS; (2) increase key stakeholder knowledge and change stakeholder attitudes, policies, and procedures; and (3) increase availability and use of WTS technologies. Key stakeholders include individuals who use wheelchairs and their caregivers, transit providers, vehicle modifiers, product developers/manufacturers, policy makers, third-party payers, clinicians, and rehabilitation suppliers.

The first goal was targeted at obtaining a complete and objective understanding of the issues and factors involved in providing safe, accessible, and usable transportation for wheelchair-seated travelers. Advances were made in documenting the incidence, extent, and nature of injuries to wheelchair riders due to motor vehicle crashes and moving-vehicle incidents, and in identifying factors that contribute to their injury risk during access and travel on private, school, and public-transportation vehicles [252–263].

The second goal was targeted to increasing understanding and knowledge by key stakeholders of the basic principles of WTS, increasing awareness of industry standards and products that comply with these standards, and changing stakeholder attitudes and policies in ways that improve wheelchair transportation safety. Progress was made in closing the gap in knowledge and awareness of WTS by providing educational chapters, websites, and tools for safe transportation [264, 265]. Changing attitudes, policies, and procedures of key stakeholders included providing actionable recommendations for changes to policies and procedures that enhance the availability and use of WTS technologies and "best-practice" in transporting people seated in all types of wheelchairs. Advances were made in publishing guidelines and position papers to guide changes in practice [266–270]. This included a RESNA position paper on WTS [271], and comments and public-hearing testimonies to the U.S. Access Board regarding the Notice of Proposed Rulemaking (NPRM) on changes to ADA Accessibility Guidelines for Transportation Vehicles. The RERCWTS also determined and reported on the state-of-science and future needs for improving WTS [272–275].

The third goal targeted increasing the availability and use of products that comply with WTS standards, including: a) innovative WTORS that offer the appropriate balance of safety, usability, and independence for different transportation environments, and b) wheelchairs and wheelchair seating systems with innovative improvements for use as seats in motor vehicles. Advances were made in product development [276–279] and related performance standards for wheelchairs, wheelchair seating, and wheelchair containment systems for use on large transit vehicles, as well as passive occupant restraint systems for people who drive while seated in a wheelchair [280–286].

##### Future directions

The RERCWTS made significant progress and had many important achievements toward improving wheelchair transportation safety. However, more work is needed to increase the safety, usability, and independence for travelers seated in wheelchairs to acceptable levels in all modes of ground transportation. Some of the most important needs for future research and development include investigating vehicle-related safety issues for drivers and front-row passengers seated in wheelchairs (e.g., hand controls, airbags, accessory storage). It is also necessary to translate safety, accessibility, and usability issues during ingress/egress of travelers seated in wheelchairs using both ramps and lifts into technology that will improve safety and accessibility. Further, there is a need to continue in-depth investigations of real-world crashes and non-crash events involving passengers and drivers seated in wheelchairs to increase the power for analyzing relationships between crash direction and other factors related to the risk of injury to occupants in wheelchairs.

Future work will also continue to update, develop, and implement WTS standards, with a particular emphasis on drivers seated in wheelchairs. An important goal is to develop performance tests and criteria for improved safety of forward-facing wheelchair occupants in low-*g* non-crash environments, especially related to seat-belt retractor technologies and their effectiveness in non-crash (i.e., below-1 *g*) vehicle decelerations. Further, it is necessary to address safety and usability issues related to the use of scooter-type wheelchairs in fixed-route and paratransit vehicles. Finally, there is a need to continue to advocate and provide support for updated ADA Accessibility Guidelines for Transportation Vehicles. The RERC on Accessible Public Transportation is providing that support in current rule making activities of the Federal government related to vehicles.

### Centers with a communication and/or cognition focus

When first established, the RERC’s primarily focused on technologies for personal mobility. Yet many issues of disability are related to sensory function, including vision and hearing, as well as cognition. In this section, we review the current RERC’s focused on rehabilitation technologies that address needs related to communication and cognition.

#### Augmentative and alternative communication

##### Need and rationale

“There is no typical person who uses AAC. They come from all age groups, socioeconomic groups, and ethnic, religious, and racial backgrounds. Their only unifying characteristic is the fact that they require adaptive assistance for speaking and/or writing because their gestural, spoken, and/or written communication is temporarily or permanently inadequate to meet all of their communication needs” [287].

In the United States, there are more than 4 million individuals (over 6% of the U.S. population) with complex communication needs who could benefit from augmentative and alternative communication (AAC) [288]. The population spans the age spectrum, disability categories (developmental, acquired, and degenerative), cultural/ethnic backgrounds, and socioeconomic classes. The AAC-RERC was formed to provide a national center with a focus on advanced engineering research and development of innovative technologies and strategies addressing those with complex communication needs.

In the early 1980s, microprocessor-based AAC devices began to appear spawning a new industry. By the mid-1990s, these dedicated AAC devices started employing mainstream computer operating systems thereby joining the digital revolution. At this time, communication was mostly about face-to-face interactions, talking on the phone, gestures, and “typing” using text or graphic symbols. AAC technologies produced synthesized/digitized speech that was not very intelligible and AAC devices were clunky, difficult to use, and challenging to learn [288].

There were some user populations that were well served by the AAC technology of the day, while others, such as young children, individuals with significant cognitive and linguistic challenges such as aphasia, autism, traumatic brain injury, and individuals with severe motor impairments, such as amyotrophic lateral sclerosis, were not [289–292]. AAC stakeholders expressed frustration and concern about the extensive learning and cognitive demands AAC technologies placed on people with disabilities and their families, as well as on the educators and healthcare providers who were trying to help them. Most AAC technologies at the time were not research based and, thus, they were not maximally effective for many individuals with complex communication needs.

In 1998, the newly formed AAC-RERC partners chose to focus on key functionality and usability features that were missing from AAC technologies, which resulted in many population groups unable to use them.

##### Advances

In advancing AAC technologies, the AAC-RERC follows two principles. The first principle is that individuals with complex communication needs who rely on AAC technologies and their family members are included in all aspects of AAC-RERC activities [293, 294]. Second, the AAC-RERC undertakes projects that are of crucial importance to the AAC field but were not being addressed by other entities. For example, the AAC-RERC activities do not focus on work in speech synthesis/recognition, eye gaze technologies, battery life, mobile technology platforms, and cellular technology, because large corporate and research entities are already working on these areas.

The AAC-RERC instead has focused on: (1) increasing the learnability and usability of AAC technologies for young children with complex communication needs and for people with cognitive and linguistic challenges (aphasia, traumatic brain injury, autism) [292, 295, 296]; (2) developing new AAC interfaces that are easy to learn and use and address the needs of people with limited movement and cognitive challenges [289–291, 297–301]; (3) improving literacy skills development, employment outcomes, and the ability of individuals to take on preferred social roles in their communities [302, 303]; and, (4) improving access to the world [304–307].

Over time, the AAC-RERC has produced a multitude of technologies and knowledge that have contributed to the growth of the next generation of AAC devices and stakeholders [288, 307]. Highlights of some of those contributions are (see also Table 2): widespread adoption of AAC design features (visual scenes; navigation/ organization/color features) suitable for children and adults (Dynavox, Prentke Romich Company, ABlenet, AMDi, IGEL, Kompaniet); free downloadable templates at aac.unl.edu; and increased availability of access technologies for people with limited movement (e.g., SafeLaser: Zygo, InvoTek).

##### Future directions

Although AAC interventions and technologies have a positive impact on the communication and participation of individuals with complex communication needs across all age groups, disability areas, and environments and are no risk to speech development or recovery; the benefits have not yet been maximized. Many individuals with complex communication needs continue to struggle to attain communicative competence to actively participate in their families, schools, worksites and communities. And, there are still groups of people (severe aphasia, dementia, and developmental disabilities) whom AAC technologies do not support well.

While face-to-face interactions are essential to life activities, technological developments such as social media, distance communication, and virtual access create new opportunities and challenges. It is essential these new technologies be accessible to people with disabilities and do not exacerbate disabilities.

Research and development efforts are needed that focus on people with complex communication needs and their use of newer technologies. The field would benefit from inter-professional collaborations in areas such as natural language processing, cognitive science, usability and computer science to (1) help support qualitative and quantitative changes in language skills over time, (2) improve working memory, dual task capabilities, and visual cognitive processing, (3) address adaptive access for people with language, physical, and cognitive disabilities more creatively, (4) evaluate consumer performance and needs across multiple social and technical contexts, and (5) develop and evaluate multi-modal access strategies. Other areas need to address access to mainstream/ universal technologies using AAC devices for people across the life span, including virtual social networking resources, and mobile devices.

Despite progress in AAC, at the 2012 State of the Science Conference, Michael B. Williams, an early adopter of AAC technologies in the 1970s and an AAC-RERC partner, warned: “Computer technology used to be thought of as the great “equalizer”; now I feel people with disabilities are in danger of being shut out by these added ‘features’ that can be utilized by the public at large, but are frustratingly useless to people with significant disabilities.” Michael went on to express concerns about gesture-based technologies and the use of voice recognition in everything, including television sets, warning the field to remain “ever vigilant”. New technologies can have both positive and negative implications for people with complex communication needs and other disabilities…and we must be wary of the digital divide.

#### Cognitive technologies

##### Need and rationale

Individuals with cognitive disabilities have been marginalized for many years by society’s unwillingness to include them – within local communities, educational systems and workplaces – in short, into the very fabric of society. While technology undoubtedly can play a vital role in decreasing this marginalization, there have been few attempts over the years to produce a coherent and sustained approach to identifying and ameliorating barriers for persons with cognitive disabilities through the use of technology [308]. Until recently, when the term “technology” was used in conjunction with “cognitive disability,” it most likely referred to an assistive technology device. Use of assistive, or even mainstream commercial technologies, by persons with cognitive disabilities has lagged substantially behind all other disability groups for many years [309–312].

NIDRR awarded its first RERC focused on advancing cognitive technologies (RERC-ACT) in 2004. At that time, data recorded of assistive technology utilization by persons with disabilities did not even include the then 21.3 million persons with cognitive disabilities as a reportable category. In fact, few studies at that time addressed the extent of technology utilization or potential barriers to access to technology for persons with cognitive disabilities [311–315].

Ten years ago, commercially available cognitive technologies, with just a few exceptions, tended to focus on lower-tech solutions with minimal emphasis paid to the use of high-tech technologies to facilitate full inclusion at home, school, work or play [309, 316–318]. There was also a somewhat pervasive attitude that persons with cognitive disabilities would benefit much less than persons with other disability types in the use or implementation of technology [310, 312, 316]. Research studies available tended to be single-case design or studies with extremely small populations [319–322]. Accessibility to Information Communication Technologies by persons with cognitive disabilities and standards were mostly ignored and/or determined to be “too hard” to address [323–326]. In short, few agencies, research labs, or organizations expressed more than a passing interest in researching and developing cognitive technologies. In terms of commercial mainstream and assistive technology, smart phones were just being considered; context aware sensors were in the very early stages, downloadable disability-related apps, the cloud, and, the “Internet of things” were not available [326–330].

##### Advances

Awareness of the benefits of technologies for persons with cognitive disabilities has changed dramatically in the intervening years. In large part, this is due to the graying of the world’s population and the increasing numbers of persons living and working much longer than ever before, many with acquired or organic cognitive and other functional impairments. IBM, Blackberry, Anthem Memory Care, ATIA, AbleNet Technologies, and other commercial partners and organizations have engaged with our RERC-ACT, expanding their and the RERC-ACT research and development activities.

**Fig. 7:**
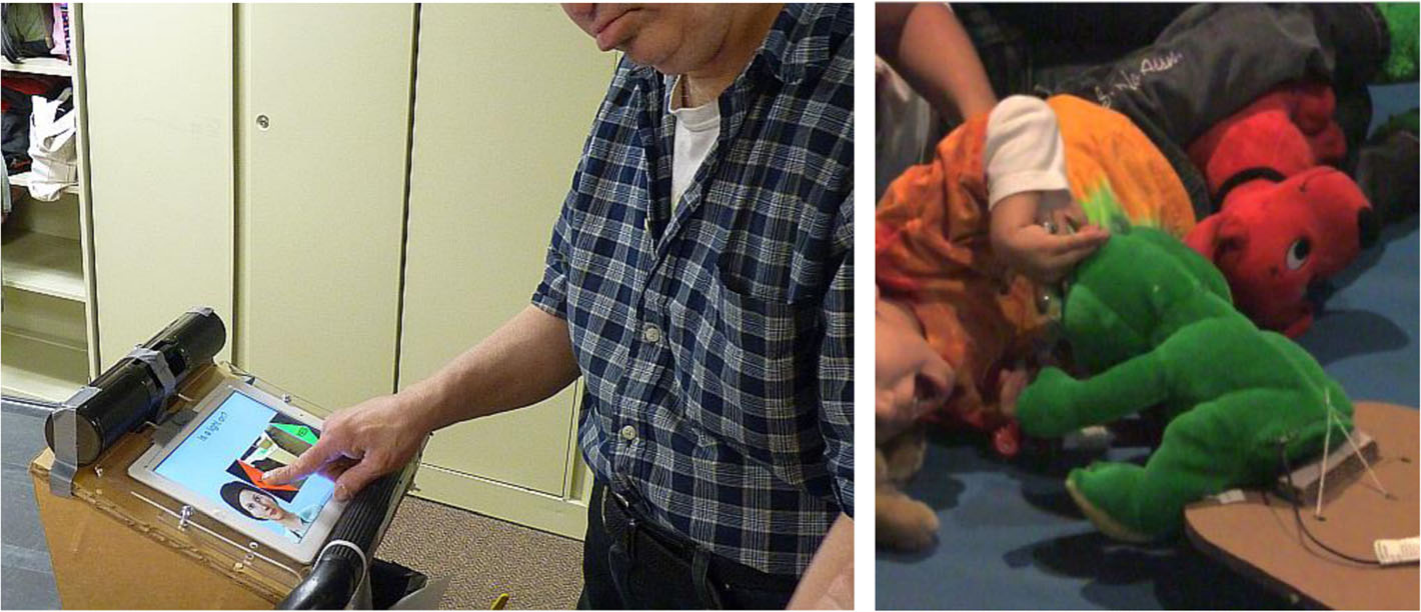
Example of a cognitive technology: an animated agent providing non-linear, context-aware job coaching. Right: A boy with cerebral palsy interacting with a social assistive robot

The RERC-ACT has helped dispel the notion that persons with cognitive disabilities cannot benefit from technology through worldwide dissemination of information and aggressive work with emerging sensor technologies and platforms focused solely on the needs of this population [331], particularly in the area of Social Assistive Robotics for children and workplace accommodations for adults with cognitive impairments (Fig. 7). A social assistive robot was developed as a research tool with the goal of engaging children with cerebral palsy. Interaction with the robot was observed and compared to the child’s engagement with a traditional switch activated toy. A nonlinear and contextually aware prompting system was developed to assist workers with intellectual and developmental disabilities perform factory assembly tasks [332, 333]. In this system prompts were delivered by an animated agent. A mobile-based vocational skill building coaching technology for people with cognitive disabilities was also developed, evaluated and found to be a useful tool [334]. These results have paved the way for the development projects of the current RERC-ACT; development of a nonlinear and context aware automated job coach for warehouse order fillers with intellectual and developmental disabilities. The RERC-ACT is pioneering more complex and rigorous methodologies with significantly larger subject populations. The Product Testing Lab has embraced testing of emerging and new technologies such as smartphones, tablets, and wearables.

##### Future directions

This is an exciting and highly dynamic time to engage in work with cognitive technologies; in large part because we are becoming a nation heavily populated by older adults [335–337] By 2030, 19% of the US population will be aging [338] with an estimated prevalence of dementia among individuals aged 71 and older at 13.9% [339].

More infants with cognitive disabilities are surviving and living a full life span. From 1990 to 2012, the world’s neonatal mortality rate fell from 33 deaths to 21 deaths per 1,000 live births [340]. Medicine is also helping trauma victims, many with cognitive insults, survive. An estimated 19% of veterans return home with a traumatic brain injury (TBI) [341]. In the U.S., 1.7 million civilians experience a TBI annually with a significant number retaining permanent disability. Costs for TBI are an estimated $60 billion per year [342, 343].

The number of adults with intellectual and developmental disabilities age 60 and older (currently >$56 billion annually) is projected to double by 2030. Research indicates if appropriate personalized supports are provided, almost all individuals with intellectual and developmental disabilities have improved life outcomes. Many adults can live independent, productive lives with support from family, friends, environmental adaptations, and access to appropriate technology solutions [344].

Future directions must encompass development of cognitively accessible medical and commercial mainstream technologies. Researchers and developers should focus on emerging technologies such as context-aware sensors, apps, social assistive robotics, and standards development to expand the continuum of independence currently visualized for others. We must develop evidence-based research tools and methodologies taking advantage of the expanding knowledge-base created by scientists around the world.

Despite the potential of emerging technologies to assist persons with cognitive disabilities, there are significant practical challenges in the commercialization process, including adoption of industry standards, reduction of consumer abandonment rates, and design and development of useful products [345]. Innovative engineering approaches, effective needs analysis, user-centered design, and rapid evolutionary development are essential to ensure that technically feasible products meet the real world needs of persons with cognitive disabilities.

#### Low vision, blindness and multisensory loss

##### Need and rationale

The blindness RERC has been responsible for many developments that are now taken for granted. This includes the first development of Tactile Vision Substitution Systems for displaying tactile images on the skin [346–348], leading to vastly increased knowledge of how to take advantage of the tactile sense and ultimately leading to devices such as the BrainPort of today [349, 350]. Other early contributions included some of the first popular devices to help blind people with specific tasks such as liquid level indicators, auditory light probes, and an array of audio-tactile output solutions to make jobsite tools and instruments accessible for blind employees in industry [351, 352]. These even included an Auditory Oscilloscope to enable a blind technician to observe and measure electrical waveforms. Techniques and training materials were developed for blind technicians, enabling them to do their own electronic circuit design and soldering [353]. The Smith-Kettlewell Technical File [354] emerged as the only technical publication by and for blind technicians and hobbyists.

With the advent of digital technology and the first personal computers, this RERC developed the first speech modules used in elevators, microprocessor-based talking tactile-haptic educational games for blind children [351], the first braille TDDs (Telecommunications Device for the Deaf) for deaf-blind users [355], the first touch-tablet based computer access system for blind users [356], and a robotic fingerspelling hand for deaf-blind communication [357]. Digital speech and infrared technologies were combined to develop and refine the pioneering “Talking Signs” navigation system for blind pedestrians [358, 359] which spread to many locations around the world and inspired a legion of other related systems.

##### Advances

The presence of the RERC within the Smith-Kettlewell Institute over a long period led to a steady accumulation of clinicians, scientists and engineers (blind and sighted) in related areas of research and with supplemental funding from other sources. These synergies eventually produced the largest non-profit center of research expertise on blindness and low vision in the world. Research into partial vision loss, including screening and assessment of function, was greatly expanded during this time, building on the pioneering development of the rapid “Sweep VEP” (Visual Evoked Potential) to enable assessment of vision impairments in infants and pre-verbal children [360]. Photorefraction methods were perfected for visual screening of young children by merely taking a photograph and having it analyzed [361]. For adults, numerous chart-based tests (the SKILL card [362], Colenbrander Low Vision Acuity Chart [363], SKRead Test [364], Colenbrander Mixed Contrast Test [365], etc) were developed as fast and clinically practical ways of better measuring visual impairment and function. The Multi-Focal EEG system [366] was developed to provide objective assessment of vision function at hundreds of locations on the retina simultaneously. The underlying technology was applied to develop the first brain communication interface for severely disabled individuals with locked-in syndrome [367].

**Fig. 8:**
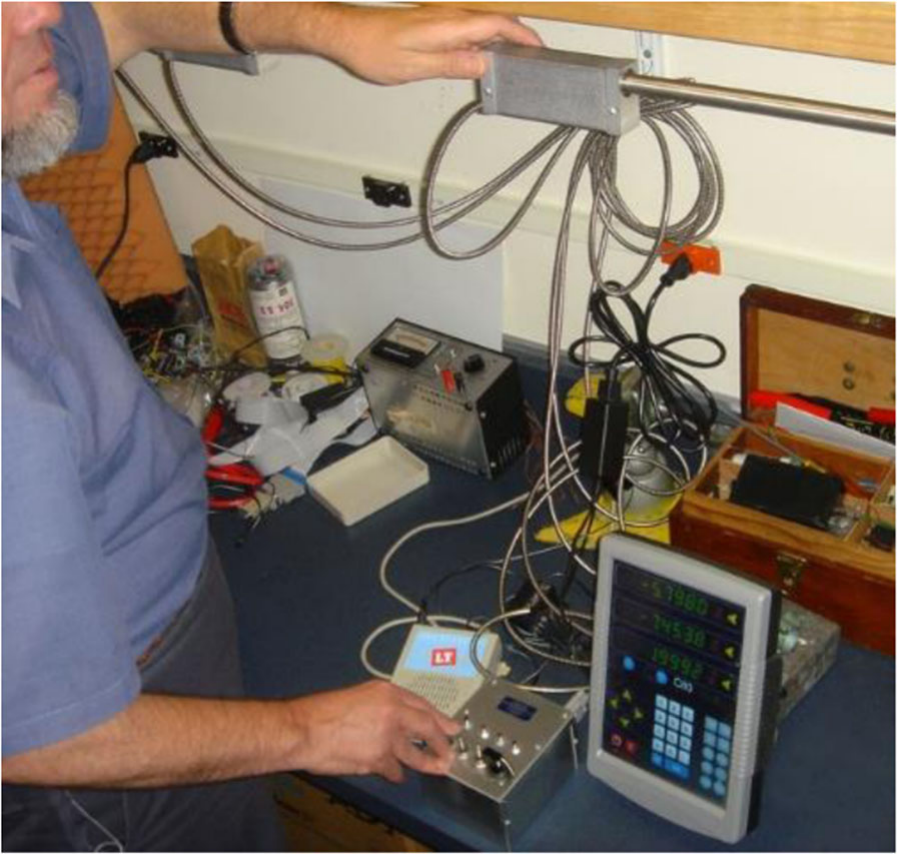
Right: Tom Fowle, Smith-Kettlewell RERC engineer who is blind, building and testing an accessible CNC milling machine interface for a blind machinist

Steadily improving digital technologies enabled the RERC to develop, or facilitate development of, the first accessible mass transit fare machines, the first accessible building entry system, and talking interfaces for computer numerical control (CNC) machines (Fig. 8). Software tools were developed and made available to enable a blind person to access Matlab [368]. Increased computing power in portable devices enabled us to pioneer the application of computer vision technology to solving problems faced by blind people such as reading displays and signs or orienting to a crosswalk [369–372].

During the rise of the Internet the RERC was at the forefront of efforts to ensure accessibility for blind users, with staff members serving prominently on several working groups within the W3C Web Access Initiative. Meanwhile we have harnessed Internet technology for numerous projects such as Tactile Map Automated Production (TMAP, [373]), a system to allow blind users to obtain custom tactile maps of any desired area in the US, and a crowd-sourced solution for providing video description [374]. Other recent projects include the adaptation of “Smart Pen” technology to provide audio-tactile access to graphics [375], investigations of the impact of and interventions for dual sensory loss in specific tasks [376], development of computerized low vision tests, and applications of visual evoked potentials for assessment of infants with common eye diseases such as Retinopathy of Prematurity and Cortical Visual Impairment [377, 378] to better inform rehabilitation interventions.

The presence of noted experts in many blindness-related fields within the RERC has facilitated our service in numerous government and industry standard-setting activities, including ANSI infrared signage standards, the Daisy Consortium for electronic book standards, the Social Security Administration, American Medical Association and United Nations standards for disability [379], and the redesign of US paper currency to facilitate use by visually impaired consumers, to name a few.

##### Future directions

During the coming years, the environment in which blind and visually impaired people live will change in ways that as yet we cannot predict, but we know the continuing advances in mainstream technology will introduce new challenges in accessing the information so conveniently available to the sighted mainstream. At the same time, the changing population in terms of types, degrees and combinations of sensory impairments will complicate the interaction between the person and the environment. Changes in the nature and informational demands of both the workplace and the community in which we live will present us with new questions to be answered about the optimal strategies for matching abilities to the requirements of education, work and community living.

#### Mobile technology to support health self-management in adolescents with disabilities

##### Needs and rationale

When transitioning from childhood to adulthood, adolescents and young adults face many challenges that are magnified by having a disability, impairment, or chronic medical condition. With regard to health management, adolescents are attempting to transition from parental management to self-management and independence [380]. This can be challenging both because they lack knowledge and skills to anticipate and avoid secondary health complications, and because they may have impairments in cognition, including executive dysfunction, that make it difficult to complete necessary tasks [381]. Few healthcare systems assess the ability of adolescents (or any patient) to accomplish key activities so that information and training can be tailored to that patient’s particular level of education, awareness, or functioning. In addition, while evidence-based, self-management approaches have existed for decades, they are most often the subject of research or public health interventions rather than integrated into interactions with healthcare providers. Technology offers new opportunities to develop and implement strategies to address these challenges by supporting and reinforcing healthful behaviors.

##### Advances

The *Technology Increasing Knowledge: Technology Optimizing Choice (TIKTOC)* Rehabilitation Engineering Research Center (TIKTOC RERC) began funding in 2013 as an interdisciplinary collaboration of clinicians and researchers focused on using networked, mobile systems to create tools for teaching, assisting, and motivating adolescents and young adults with disabilities to take increasing responsibility for independently managing their health within community environments.

Through our recently published *Model of Healthcare Disparities and Disabilities* [382], we have conceptualized the issues that individuals with disabilities experience as the interaction of impairment with the context in which impairment occurs – including both the environmental and personal factors that affect the severity of the manifest disability. This model, then, allows us understand mobile technology as a modifiable factor that can be tailored to enhance health and participation outcomes. This approach should also lead to increased transfer of knowledge and/or technological development beyond that used by individuals with a single diagnostic group, to applications and utilization by individuals with multiple relevant impairments.

TIKTOC RERC investigators are working to identify cognitive and motivational factors that have an impact on the ability of adolescents and young adults with neurodevelopmental conditions (NDCs) to self-manage their health. Findings from this study will be incorporated into the design of an interactive mobile application that learns to select messages and prompts to support medication management among young adults with NDCs and used to produce Guidelines and Recommendations for targeting and tailoring health self-management interventions and mobile apps in ways that compensate for executive dysfunction and address motivational factors. While this project has not yet been completed, initial results reflect significant differences between the perceived and actual abilities of adolescents and young adults with executive dysfunction to perform complex health management activities [383] and speaks to the importance of assessing and addressing these factors to create realistic health management plans.

**Fig. 9:**
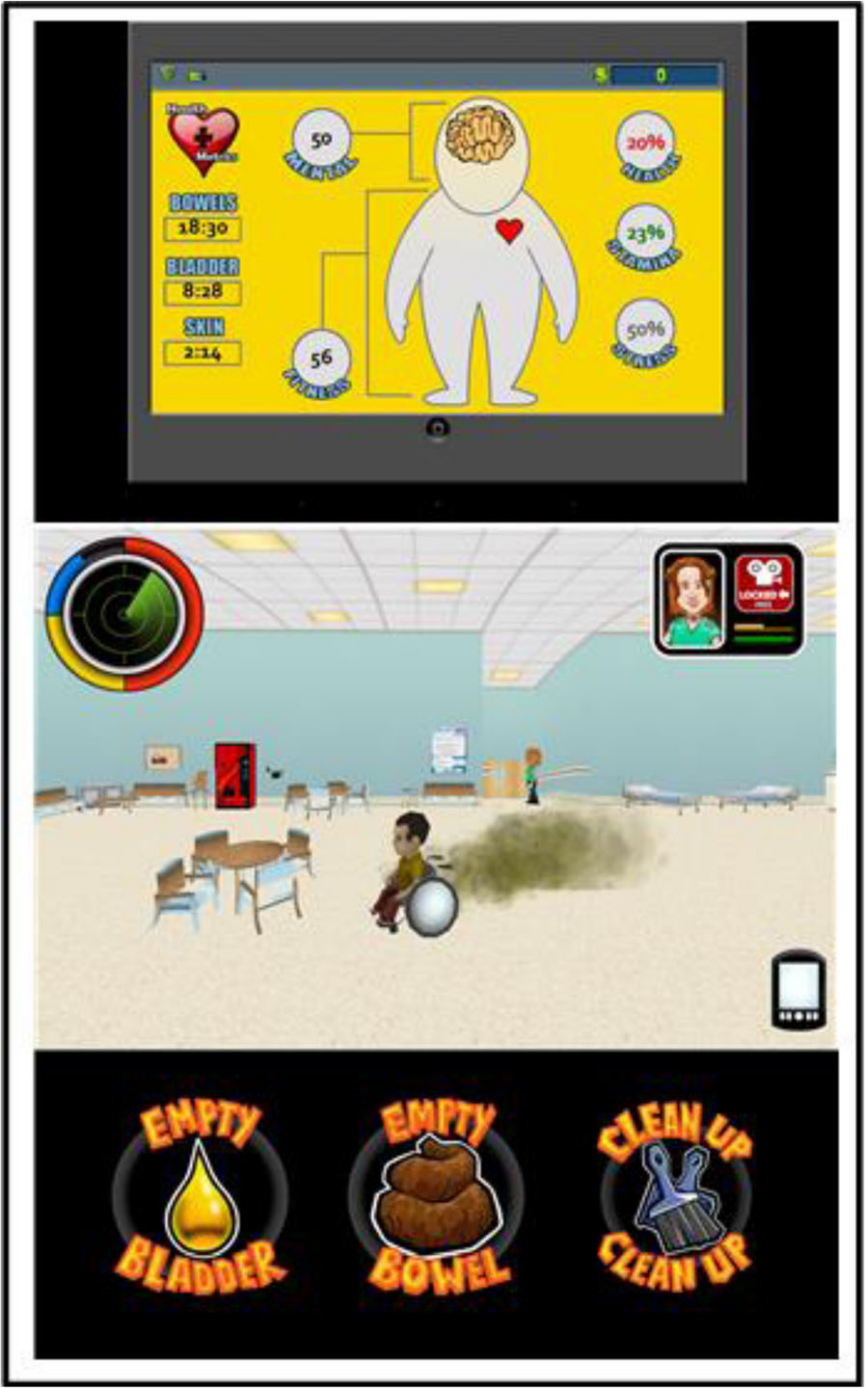
Screenshots from the Serious Gaming App *SCI Hard* illustrate within-game methods for bowel and bladder management, including monitoring (upper picture) and emptying (lower figure) as well as the possible consequences of failing to engage in appropriate self-management techniques (i.e. formation of stink cloud in middle picture).

The RERC is also supporting the final development, evaluation, and transfer of the mobile game *SCI HARD* (Fig. 9). *SCI HARD* was created to enhance self-management skills, health behaviors, and participation among adolescents and young adults with spinal cord injury through an approach that is scalable as well as accessible and engaging to this target population. Current research efforts will generate important data to determine if the serious game represents a beneficial and cost-effective complement to existing rehabilitation approaches. Already our development and dissemination efforts have increased awareness of potential ways to tailor educational and self-management training to better match the needs and strengths of various populations [384].

TIKTOC RERC investigators have developed underpinning technologies for augmenting cognition and memory to improve self-management decisions and adherence to health needs. This decision-support technology will be used in programs that help inexperienced adolescents avoid overestimating or underestimating risk so as to maximize safe, positive participation in meaningful activities and life experiences.

Finally, TIKTOC RERC investigators are working toward the development of cloud-based assessment and coaching tools to provide support for adolescents and young adults with disabilities in setting goals, measuring progress, sharing knowledge and best practices, and developing personalized training and self-management plans for themselves and family or care attendants.

##### Future directions

In the next 5 to 10 years, we believe that the key questions and challenges will be associated with how to use technology to optimize communication and linkages both between individuals and health care systems as well as between systems such as healthcare and education. As such, it will be critical to design technological interventions that support a continuum between training and cognitive orthotics. During the transition to adulthood, adolescents will incrementally take on more self-management responsibility; a fundamental opportunity that software-enabled technologies provide is adaptation to the needs and abilities of the user, where a system can challenge an adolescent to learn to be less reliant on the technology, and at the same time serve as a backstop to prevent (or contain the consequences of) self-management mistakes.

It will also be important to design or improve technology to support continuous, direct behavior measurement and to create better associated logical and interventional frames of reference for personalized behavioral interventions, as opposed to current "one-size-fits-all" treatment concepts. Similarly, treatments are now adjusted by clinical experience as opposed to data-based decisions, and both hardware and software development is needed to improve personalization.

Finally, any enhancements in technology must be matched with improvements in dissemination, adoption, and financing of innovations for routine clinical use everywhere in the US, so that even smaller agencies rapidly adopt the advances developed at engineering centers and RERCs, and the gap between research and widespread adoption is reduced.

#### Technology for successful aging with a disability

##### Need and rationale

Historically, research in disability and aging has emphasized the impact of either increasing levels of chronic illness and functional losses in late life (*aging into disability)* or aging and congenital or acquired impairments from early to middle life (*aging with a disability)*. The former has been primarily the purview of geriatrics/gerontology, and has an aging research approach (i.e., understand and control factors that affect aging) more than a disability research approach (i.e., understand and compensate for factors that affect disability). In contrast, the latter, which has been the interest and focus of rehabilitation engineering and RERCs under NIDRR’s priorities, has focused primarily on understanding the consequences of life-long impairments in old age and early-onset of aging due to disability.

Although both approaches are important, each only addresses half of the aging problem in that they both overlook the 29.5 million Americans aged 21-64 who are now growing older with a long-term impairment or disability [385] and who will likely experience newly acquired and pervasive age-related functional losses, comorbidities and secondary conditions [386–391]. For these individuals, the additive effects of age-related conditions may mean the difference between their current impairment or disability and aging into disability or multiple disabilities, respectively.

There are few published studies about the effects of rehabilitation interventions for people with age-related deficits in function among the population of people aging with impairment or disability. Evaluation of existing rehabilitation engineering interventions, usability testing, and research devoted to increasing the availability of technologies for this population is lacking; therefore little scientific evidence exists with which to inform rehabilitation engineering practice. Thus, despite comprising the majority of the population of seniors with disabilities, individuals who are experiencing age-related limitations beyond their primary impairment/disability are also the most underserved and understudied target population. Our goal is to influence rehabilitation engineering practice by assessing the impact of age-related changes on the activity and participation needs and outcomes of people growing older with impairments and/or disabilities.

##### Advances

Working within a universal design paradigm that drives all RERC activities, RERC TechSAge serves as a catalyst for a major shift in the understanding and design of home and community technologies for people aging with impairment and disability. Our mission is to conduct advanced rehabilitation engineering research and development to prevent, minimize or reverse the disabling effects of age-related losses and contextual factors on the independence, health and participation of people who are aging with chronic conditions or long-term impairment. Currently in year 2 of the grant at Georgia Tech, RERC has already made important strides in setting the foundation for strategic R&D projects to understand and support the experience of individuals aging with disability.

Research activities are underway to provide converging evidence necessary to design integrated technology supports for seniors aging with disability. Specifically, RERC TechSAge is developing an evidence-based taxonomy of user needs, stratified by functional loss; identifying needs and predictors of interventions for home-based tasks; and demonstrating feasibility of using functional performance data to predict task performance within and across activities. The RERC has developed a participant registry of people aging with disability to provide efficient study-specific recruitment for projects as well as a Minimum Assessment Battery, to standardize measures across all TechSAge participants. A large-scale database has been developed to integrate both assessment and project-specific data to identify patterns of ability, performance, and technology needs.

**Fig. 10:**
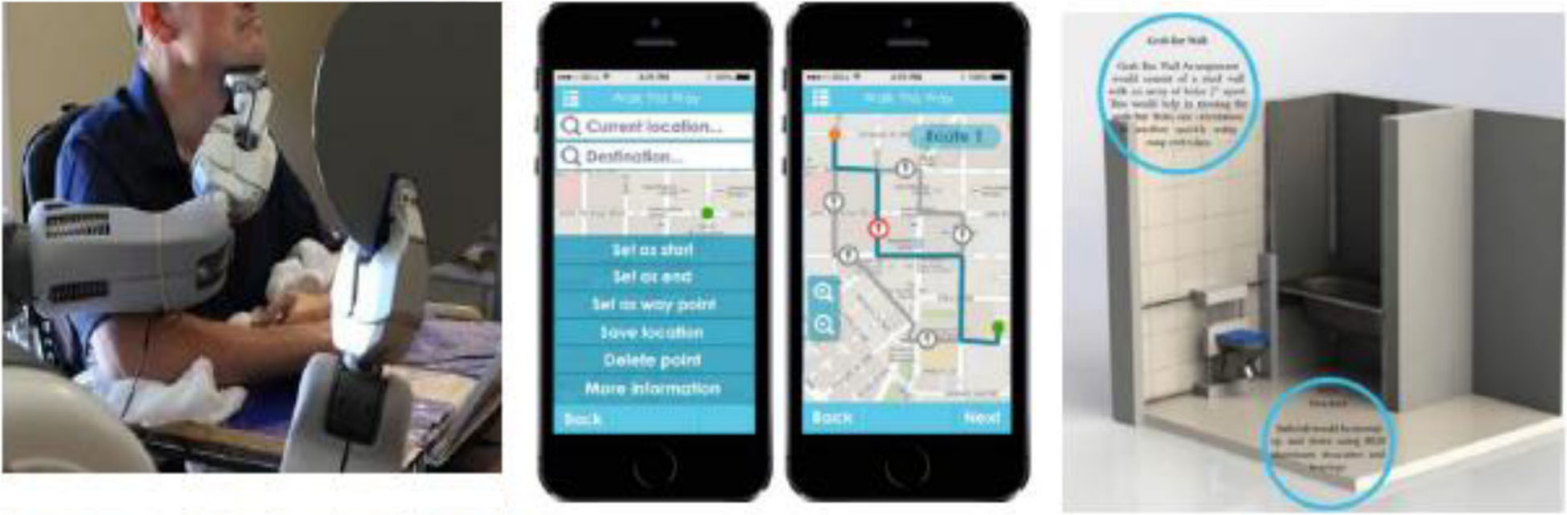
RERC TechSAge Projects. Left: The Mobile Manipulator Robot demonstrates how a robotic assistive system is helping Henry Evans, a stroke survivor with limited mobility, with routine shaving tasks; Middle: The ALIGN app enables seniors with mobility disability to select routes based on environmental preferences and characteristics and accessibility needs; Right: The SmartBathroom laboratory in GA Tech’s AwareHome will permit rigorous testing through mechanized fixtures and grab bars that are adjustable in multiple planes

Development activities have short-term and longer-term outputs and outcomes. Our app development to promote successful aging with disability will advance the rapid and cost-effective deployment of technologies through software development and evaluation [392]. First versions of the route planning and cognitive gaming apps have been developed. In addition, the cognitive gaming project has developed cloud-based solution for high-precision player data logging, a web service for streaming to a database, and hundreds of new levels informed by our research. The SmartBathroom project is currently in the construction phase of a state-of-the-art, context-aware, fully automated bathroom with continuous monitoring of a user’s functional status (e.g., gait, balance, posture) and task performance (e.g., toilet and tub transfers) to eventually develop algorithms that will synchronously adjust environmental features (e.g., grab bars, fixtures) based on user needs [393]. Finally, working with an individual with ALS, the mobile manipulator robot project has developed and installed a robotic bed in the participant’s home designed to assist with body positioning for various reach tasks; and has refined the web-based interface to facilitate the participant’s control of the robot [394, 395] (Fig. 10).

##### Future directions

The RERC’s approach to aging with disability is only beginning to scratch the surface of understanding the problems and developing solutions for our target populations. In fact, the premise of much of the basic research undertaken by the RERC is to identify the set of questions to be addressed by future RERC efforts.

The current RERC development projects have been designed to test usability and utility with pilot data on effectiveness. Longer term there is a need for translational research of these evidence-based interventions to identify intervention efficacy on health, activity and participation of people aging with disability. In research there is the need to examine the use of technology supports and changes in adoption strategies over time as older adults with impairments and some disability age into either disability or greater disability. Because much of the RERC’s target audience has historically been underserved by traditional assistive technology and rehabilitation engineering interventions, it is important to examine indigenous, individualized solutions that will scale up to customizable universal design solutions. Finally, there is need to understand and develop universal design smart interventions that not only compensate for disability, but are capable of effecting behavior change that enhances the acceptance and effectiveness of the interventions.

#### Universal interface and information technology access

##### Needs and rationale

The UIITA-RERC began in the 1980s as the RERC on “Functional Information Access & Transfer.” The focus then was on providing access in a way that was transparent to the computer, such that the computer could not tell that the users with disabilities (using special input devices) were not using the computer’s standard input devices. From his work the RERC expanded over time to include access to public access terminals (kiosks, ATMs, POS devices, voting, etc.), the Web, smartphones, and finally, any device with a digital interface.

In all of its work the RERC focused on moving beyond research to commercial transfer and system change. The RERC sought and seeks to answer questions, define needs, create solutions, lower the cost to make things accessible -- and to support all others (researchers and developers to clinicians and consumers) who are trying to advance accessibility. To accomplish this the RERC has worked with over 50 companies, and numerous consumer and government organizations. The work of the RERC over the years can be broadly divided into 4 areas.

*Area 1 – Transparent Computer Access (1980s onward):* Disability-oriented software in 1980 focused on special programs specially designed or adapted for people with disabilities. Because of the lack of any I/O handling in the operating system, (software directly read the I/O interfaces), providing access to mainstream software would require special hardware that directly replaced the mainstream interface device. A dual-computer approach was proposed to provide “transparent access” to all of the software (mainstream and special software) on the computer allowing the full resources of the computer(s) to be used both for the special software and for the mainstream software, and it provided full access to any mainstream software installed on the mainstream computer [396]. Both internal and external “dual computer” approaches were highlighted in Byte Magazine in 1982 [397, 398]. The RERC then moved these concepts to the market first with the KEI (Keyboard Emulating Interface) Standard and then commercial KEI’s that enabled AT users to control Apple, IBM, and Linux computers. When mice came into the picture the RERC revised the standard to cover both keyboards and mice [399] and then developed a hardware device that implemented the standard, the Trace Transparent Access Module (TTAM) [400]. It also developed a software version as an extension to Microsoft Windows [401]. Both were successfully transferred to industry, the hardware module the AT industry, and the Windows extension was transferred to Microsoft, who built it directly into Windows 95 and subsequent versions of Windows.

The RERC also worked with Apple, Microsoft, and IBM to build other access features directly into their systems. Starting in 1986the RERC worked with Apple to built access into their Apple IIe, Apple GS, and Macintosh computers. Three of the first five access features in Apple’s operating system were first developed at the RERC and then transferred; StickyKeys, MouseKeys, and SlowKeys. These were the first access features built into any standard commercial computer operating system. Simultaneously, the RERC worked with Microsoft, IBM. The RERC developed access features that were distributed by Microsoft on their driver disks starting with Windows 2.0. The RERC also created AccessDOS for IBM, which contained ten access features written by the RERC, licensed (royalty free) and distributed as a (free-of-charge) IBM product. In 1995 Microsoft first included access features as part of the standard Windows operating system. Nine of the ten access features Microsoft built into Windows 95 were features that were licensed (royalty free) from the RERC.

In parallel with this work, the RERC worked on cross-disability consumer-industry accessibility standards. The RERC developed the first set of hardware/software accessibility guidelines for computers for the White House Committee on Computer Access in 1985. These guidelines were then extended and customized to serve as the first accessibility guidelines used internally by IBM (1986), and after additional work, the guidelines used by the Information Technology Foundation of ADAPSO (ITF) and Microsoft Corporation (who first distributed the RERC developed accessibility guidelines to all of its developers, and then used them as the starting point for creating their Windows-specific accessibility guidelines). Later updated versions of these and other RERC guidelines [402, 403] were used in creating the first Section 508 guidelines.

**Fig. 11:**
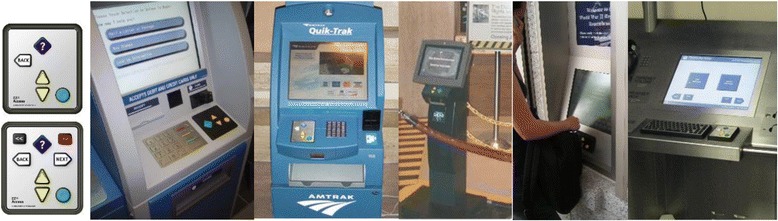
From left to right: EZ Access keypads and USPS, Amtrak, Smithsonian, US Customs, WWII memorial and Phoenix Airport use of EZ Access

*Area 2 – Access to Public Access Terminals (1990s onward)*: Starting in the early 1990s the RERC expanded its focus to include public Information technologies. Again, the focus was on enabling people with disabilities to be able to use mainstream information, ticketing, ATMs, vending, voting, and other public terminals. However, in this case, they were not personal devices, so nothing could be installed on them by a user. The strategy adopted therefore was to create a package of interface options that would enable public terminals to be used by people with as wide a range of disabilities as possible – without the user having to adapt the terminals. Initial implementations, including the kiosks at the Mall of America, were modal, requiring users to put them into one or another mode of operation that matched users’ abilities. This worked but not for less digitally adept users. Over time a cross-disability access package (dubbed EZ Access) was developed that was non-modal and easy to use and understand (Fig. 11). It provided users with the ability to operate the device and receive its output in multiple ways – similar to being able to use a keyboard or mouse to do the same thing on a computer [404, 405]. The EZ Access keypad and software interface extensions allow public terminals to be used by people who are blind, who have low vision, who have limited reach, who can’t use a touch screen, who are deaf or hard of hearing, who have cognitive disabilities, or who cannot read for any reason, as well as by anyone who would rather operate the machine from the keypad rather than the touchscreen (long fingernails, mittens,etc) [406]. The EZ Access techniques are now implemented in over 50,000 cross-disability accessible USPS Automated Postal Centers, Amtrak ticket machines, kiosks in memorials, museums, and machines in airline terminals.

At the same time, the RERC provided technical assistance to consumer groups and advocates working on ATM access, resulting in 50,000-100,000 accessible ATMs using cue and respond accessibility.

*Area 3 – the Web (1995 onward):* The Web was the second focus of the RERC that began in the 1990s. In 1995 the RERC created the first Web Accessibility Guidelines after WWW2 in Chicago [407]. In 1996 the RERC united 35 efforts that had arisen, to create the Unified Web Accessibility Guidelines, Version 8.0 of which was used as the starting point of the W3C’s Web Content Accessibility Guidelines [408]. The W3C-WAI asked the RERC to co-chair and support the WCAG working group, which it did through 2012, including development of both WCAG 1.0 and 2.0. The RERC also provided extensive research and technical support, including quantification of measures, creation of open-source test tools, and database development for the working group. WCAG 2.0 is now the international standard, not only for web content accessibility, but also as the basis for most of the software and electronic document accessibility requirements in the US, Canada, Europe, Australia and other countries.

*Area 4 – Assistive Technology (1980 onward):* Throughout its existence the Trace RERC has worked closely with the assistive technology industry, carrying out research for them, feeding R&D prototypes and production designs to them [409–411] and developing master listings, databases, and directories of assistive technology that were used as central references for clinicians [412–414].

##### Advances

Since the early 2000s, a perfect storm has been brewing. Society as a whole is moving to technology in all aspects of life, (education, employment, communication, healthcare, civic participation, etc). People who can’t use technology can no longer avoid it. Yet we do not have assistive technologies or access strategies for all, and the proliferation of platforms means that even those that have AT, do not have it on all the devices they encounter and have to use in daily life. And the funds to provide access are decreasing as the number needing access increases.

To address these and other related issues the RERC brought together an international coalition, the Raising the Floor Consortium, to seek answers [415]. It found early on that the problem could not be met with the current accessibility ecosystem, which was only reaching between 3 and 15% of those who needed special interfaces. The RERC proposed the development of a Global Public Inclusive Infrastructure (GPII) that would 1) make it much easier for people facing barriers to ICT use due to disability, literacy, digital-literacy, and aging to find what features or solutions they needed; 2) make it possible for them to use that information to cause any ICT they encountered (computer, phone, ticket machine, TV) to instantly change its interface into one they could understand and use; and 3) make it much easier for developers of all types (large, small, AT or mainstream) to explore, create, test, and market new and better solutions internationally, including for very-low-incidence solutions.

The concept spread rapidly internationally, with projects now in process in the US, Canada, and Europe [416]. Over 50 companies and organizations, and over 100,000 individuals have now joined in the effort. The focus is now on secure necessary funding and moving the GPII from research to real-world implementation and international availability [417].

##### Future directions

Going forward the RERC is focused in three key areas. First, we will address the issues created by the move by society to all digital technologies, everywhere, in every activity. Second, and related, there is a great need for everyone to be able to understand and use the digital interfaces they are encountering, across devices, operating systems, and environments. Third, we will develop ways to reduce the cost and effort needed to make things accessible so that mainstream and assistive technology vendors will be able to address everyone including the tails and tails of the tails.

The GPII is designed to create an infrastructure to make this easier, but it is not the solution – just a necessary substrate for it. Much more work is needed in cognitive, multiple-disability, and non-technology adept portions of all disabilities. Solutions that will work in clouds, across platforms, and in homes are also challenges as are solutions that work across all of the digital interfaces, devices, and platforms encountered are still out of reach.

#### Wireless technologies

##### Needs and rationale

The Wireless RERC launched in 2001, when wireless technology was on the cusp of a revolution. WiFi was a novelty and the “cloud” was still largely a dream. Rudimentary internet access was available on a limited number of “(not-so)-smart” phones. Social media was limited to email and nascent SMS text messaging. Bluetooth standards were in development; commercially available, Bluetooth-enabled devices were in the future. Hearing Aid Compatibility requirements for mobile phones was a concern of the Federal Communications Commission and industry. In 2002, Microsoft released its Windows Mobile operating system, which supported third party screen readers and was the leading solution for blind users for most of the decade. By 2004, 68% of people with disabilities owned a wireless/mobile device.

According to 2007 Survey of User Needs data, 85% of people with disabilities owned a wireless product. By 2013, wireless device ownership increased to 91%. Inclusion of critical accessibility features led to product loyalty among disability groups. Some companies have addressed accessibility concerns with their own solutions. The Apple iPhone, with its icon-based touchscreen interface and robust ecosystem of mobile wireless “apps”, revolutionized the smartphone and its capabilities. In the Deaf community, smartphones, tablets and other wireless devices have quickly become necessary technologies. AAC users use tablets at substantial rates, due to barriers in communicating synthetic speech via smartphones. Digital assistants, voice inputs and outputs are major facilitators for people with vision loss and limited upper extremity function. With millions of free apps consumers are increasingly turning to recommendation engines, friends, social networking or advertising to discover mobile applications rather than sorting through available apps. This presents a challenge for app developers, even when the app is truly unique and necessary for people with disabilities. Regarding social media, trustworthiness of an information source (especially in emergencies) is a critical barrier/facilitator to use. Among individuals with disabilities, they are more apt to follow “trustworthy, credible” organizations online and believe the information they receive. Hurricanes Katrina, Rita and Sandy, highlighted the vulnerability of mobile wireless communications. As a result, effective and *inclusive* wireless emergency communications became a top priority for the FCC and DHS.

##### Advances

**Fig. 12:**
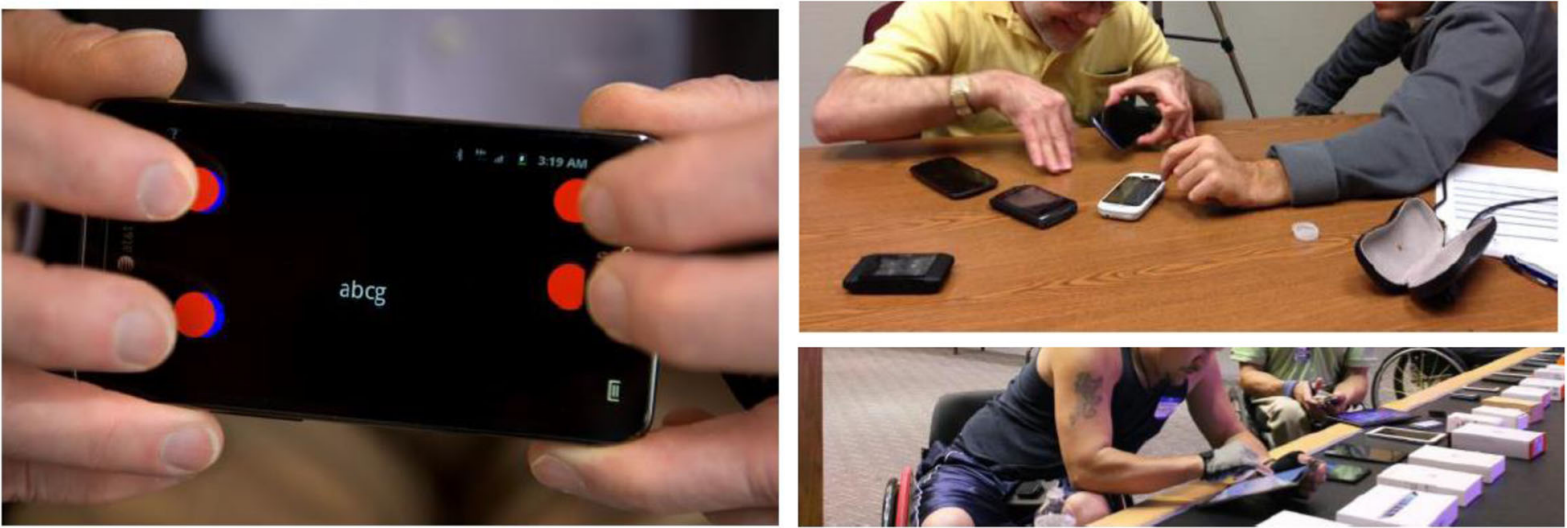
Left: BrailleTouch smartphone app developed with support from the Wireless RERC's App Factory development project. The app allows the user to type braille directly on their iPhone or iPad, and send text messages, tweets, and emails from the touchscreen braille keyboard. You can also copy text that you enter using braille and paste it into any other app on your iPhone. Upper right: Helping consumer with cerebral palsy choose new smartphone. Lower right: Wireless RERC team conducting hands-on training session on built-in accessibility features in contemporary smartphones and tablets

During its first grant cycle (2001-2006), the Wireless RERC made several important advances (Fig. 12). It pioneered a Consumer Advisory Network (CAN) and Survey of User Needs (SUN) to promote accessible products and devices and ensure that the Center’s work addressed user needs. The CAN grew to over 1,400 consumers with disabilities throughout the US. It also pioneered the use of the Blackberry platform as a data compiler (e.g. remote monitoring of weight shifts in manual wheelchair users) and providing email access to users of AAC devices. This project prompted industry-leaders to add a cell phone port to some of their AAC devices. The Mobile Accessibility Guide (MAG) prototype included a personal digital assistant to identify and record accessible and non-accessible locations. The MAG provided the proof-of-concept for today’s crowd-sourced information sites and apps for accessible public places. It also was a pioneer in “wearable” computing, using a heads up display, one-handed “twiddler” keypad, and gesture recognition inputs. This work was one basis for the development of Google Glass. The team also pioneered early, wireless remote monitoring, and received a patent for a miniature wireless accelerometer.

During its second and third funding cycles (2006 to present), the Wireless RERC made several important advances. It received supplemental funding from NIDRR in 2008 to test the FCC wireless alerting system parameters, resulting in establishment of accessible emergency alerts delivered in multiple modalities to mobile devices. Filings in 2008 contributed to new changes in regulations “we amend the FCC rules to ensure that persons with disabilities have access to public warnings.” It developed a wireless system which interfaced with public captioning systems to provide captions for recorded and live events on a user’s mobile device. The system was piloted in Redskins stadium in 2009 and used in the Super Bowl at Cowboys Stadium in 2010 [418]. The captioning system was licensed to the Monterey Bay Aquarium, University of West Georgia, and Dallas Cowboys. It pioneered the App “Factory” concept of rapid development of discrete technology applications that work on contemporary smart devices [419]. Apps for blind/low vision users included Braille readers, currency identifier (received NIDRR supplemental funding), and apps for those with cognitive or communication issues including talking photo diaries. Since 2011, eleven mobile apps have been released and have accumulated over 500,000 installations. The external alerting interface device enabled people with sensory disabilities to be aware of incoming wireless emergency alert messages. The disability community and Federal government agencies such as DHS, FEMA, FCC, and state emergency management entities’ endorsements have led to the development of a portable, traveler’s version.

##### Future directions

Mobile, wireless, cloud-based and wearable technologies are converging into an Internet of Everything, allowing for innovative solutions for accessibility. How can engineers and clinical technician’s partner in this environment to ensure the creation of accessible solutions? What are the consequences of the migration to mobile broadband and next generation technologies on legacy accessibility services (e.g.TTY/TDD)? Wearable technologies can be developed to support wellness, safety and independent living. How can they also support education and employment of individuals with disabilities? Can ubiquitous sensing and cloud-based data analytics provide meaningful support to research and development efforts in next generation wireless technologies? As innovative wireless technology increases, will the personalization of technology reduce the distinction between mainstream and assistive technologies? That is, will mainstream technologies effectively address personalization options to include people with disabilities and reduce the need for specialized assistive technologies? What technology/engineering components can be maximized to realize low-cost “smart” environments (public and private) to enhance independent living? Will advances in display, control technology and miniaturization lead to more robust accessible wireless solutions, for example, enable instantaneous translation of spoken word to sign language or text? How can location-aware technologies and Geographical Information Systems (GIS) be leveraged to increase the safety of individuals with disabilities during emergencies?

### Centers with a focus on rehabilitation therapy and exercise

As well as the expansion of the original RERC research portfolio from personalized to societal mobility issues and to communication and cognition, a third trend has been to expand RERC research into issues of rehabilitation therapy and exercise. Exercise is one of the most powerful modulators of human health and wellness, and yet many people with a disability have difficulty accessing exercise opportunities. Further, starting in the 1980’s, the substantial capacity that people with even severe impairments have for neural plasticity began to be scientifically discovered and studied, raising the question of how technology could promote optimal recovery of function.

#### Interactive exercise technologies and exercise physiology for people with disabilities

##### Need and rationale

Despite what we know about the positive effects of physical activity in improving health and function in the general adult population [420], people with disabilities remain one of the least active populations in society [421–427]. They often experience a higher number of barriers in the home, neighborhood and community when compared to the general population, restricting their options in initiating and maintaining an active lifestyle [428–435]. The multiple barriers to physical activity experienced by people with disabilities reduce their likelihood of acquiring and practicing regular physical activity routines [436]. This can lead to health decline and a cycle of deconditioning in which deteriorating physical function produces greater inactivity, further physical decline, and higher risk or severity of chronic and secondary health conditions [437–440]. Physical inactivity, combined with the presence of secondary health conditions, use of medications that cause weight gain as a side effect, and biological changes associated with aging can lead to a substantial loss of skeletal muscle mass and an increase in adipose tissue (i.e., sarcopenic obesity) in people with disabilities [426, 427, 437, 438].

**Fig. 13:**
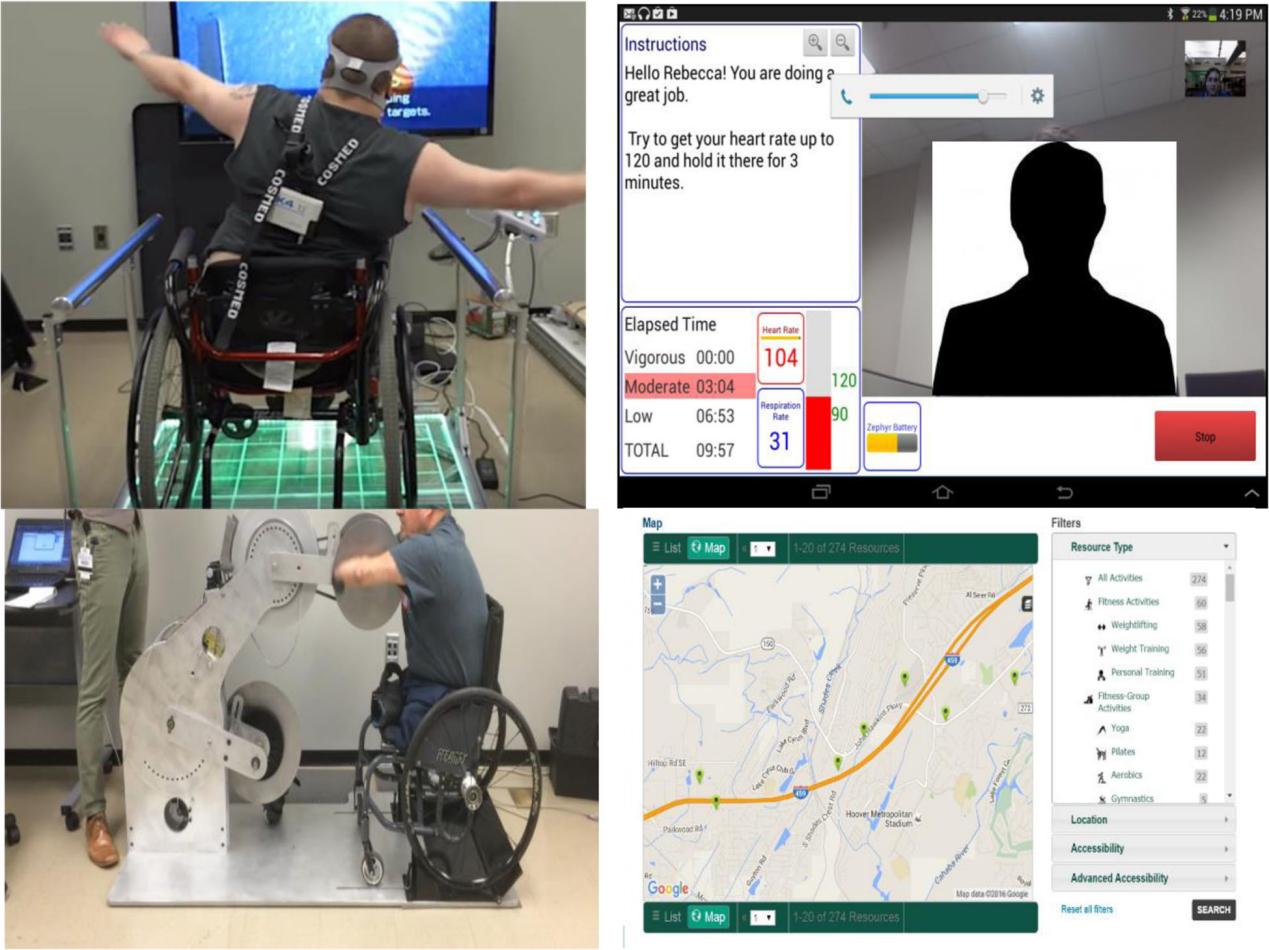
RecTech Exercise Technology Devices and Products. Top Left: Participant playing active video game on an adapted gaming controller. Top Right: Screenshot of teleexercise dashboard showing live video feed from coach, text chat with coach, and sensor data. Bottom Left: Participant exercising on a new universally designed Advanced Virtual Environment Exercise Device (AVE2D) prototype Bottom Right: Screen shot of geocoded and crowdsourced community-based physical activity resource identification system

The energy expenditure associated with participation in various forms of physical activity including fitness, sports and recreation can mitigate several of the health risks associated with sedentary lifestyles and is an important contributor to attaining and maintaining optimal health status [441]. Due to burgeoning technological advances and the ease at which they are becoming readily available for widespread use, applying new and emerging technologies may be an ideal method to support the health/wellness needs of people with disabilities. The Rectech RERC’s mission is to promote the health, function and participation of people with disabilities by a) developing new technologies or adapting existing technologies that prevent secondary conditions including reduction in obesity and deconditioning; b) increasing community participation in health/wellness activities through the creation of inclusive recreation and fitness communities; and c) reducing healthcare utilization including costly hospitalizations by providing people with disabilities appropriate, accessible and safe self-management exercise tools that empower them to maintain and improve their own health (Fig. 13).

##### Advances

RecTech has made several advances in moving new technologies and standards into industry and practice. Key products include the following.

RecTech published a set of universally designed fitness equipment standards supported by ASTM. These standards have a direct and immediate impact in promoting accessible lines of fitness equipment. Senator Harkin proposed a Bill to promote the provision of exercise and fitness equipment that is accessible to individuals with disabilities (*Exercise and Fitness Fitness For All Act*). This Bill references RecTech’s work with the ASTM equipment standards (ASTM F3021-13). RecTech will serve as the first National Standards Lab for Universal Design of Fitness Equipment. Over the last decade we have seen the number of equipment manufacturers that are designing product lines that have accessible features more than double (eg, swing-away seat, one-handed access for changing weight stacks, lighter starting loads on weight machines, better color contrast on display panels, etc.). This includes some of the largest manufacturers – Life Fitness, Cybex, Technogym, Hur and several other companies. RecTech is also developing standards for specific equipment like cycle, treadmill, elliptical and strength equipment.

RecTech also developed a Web-based intelligent weight loss/weight management system designed to promote physical activity and improve diet for youth and adults with physical disabilities and referred to as the Personalized Online Weight and Exercise Response System (POWERS). The system is currently being tested in two separate research studies. Non-disabled adults demonstrate better weight loss and weight maintenance when they use a pedometer or accelerometer to monitor daily physical activity. A first generation energy expenditure estimator App has been developed that measures energy expenditure (calories expended) in manual wheelchair users with the commercial activity monitor, SenseWear armband (Bodymedia,Inc., Pittsburgh, PA). Prior to this research, there was no way for manual wheelchair users to determine how to balance the energy equation (calories consumed vs. calories expended).

Another development is a crowd sourced online community resources mapping system called the Activity Inclusion Mapping System (AIMS). This system is designed to promote accessible physical activity by enabling the public to search for inclusive fitness opportunities through its online directory. This system, when matched with other valuable web resources such as census data, ride share services, etc. provides users and researchers with significant community access points for achieving physical activity. RecTech also developed a first generation home-based remote exercise training and monitoring system for people with disabilities (Telehealth Exercise Training for Monitoring and Evaluation, TExT-ME). In addition, a teleassessment tool (i.e., phone, iPAD, laptop) has been developed that measures the accessibility of health clubs and fitness facilities (AIMFREE) in real time and is available free of charge to professionals or consumers with disabilities anywhere in the U.S. or world. Once the audit is completed, the AIMFREE links the person (professional or consumer) to an online solutions database consisting of low and moderate cost solutions.

RecTech has also developed accessible Active Video Game Controllers for Wii Fit board and Wii gaming mat. These adaptations will open up the opportunity for many people with mobility disability to play these games equivalent to their able bodied piers. Further, a universal Advanced Virtual Environment Exercise Device (AVE^2^ D) prototype has been developed that enables both upper and lower body exercise to function asymmetrically depending on individual limb function. The device is being used with our Virtual Exercise Environment (VEE) system, which provides individuals with disabilities the opportunity to exercise in various types of outdoor VEEs (e.g., trails, parks, etc.).

Finally, a RecTech Wiki provides engineers, entrepreneurs, small startup companies, people with disabilities, etc. with design ideas for manufacturing accessible exercise/recreation products for people with mild to severe disabilities. RecTech has helped numerous entrepreneurs design accessible fitness products for people with disabilities. RecTech was also involved in the development of a training certificate approved and offered through the American College of Sports Medicine called the Certified Inclusive Fitness Trainer, which trains health/fitness professionals on how to assess and design accessible fitness facilities and implement safe and effective exercise programs for people with disabilities.

##### Future directions

People with disabilities continue to face limited options for exercise and leisure physical activity and experience barriers to taking advantage of existing opportunities (e.g., transportation, extra time needed to prepare, adapted equipment). The rapid advancement of technology requires a strategic framework and coherent context for what and how technologies can be used to promote higher levels of physical activity among people with disabilities. The RecTech model uses the metaphor of a ramp to symbolize the importance of four critical Research and Development (R&D) domains framed under the heading, Restoring Access, Mobility and Performance. The model emphasizes the logical sequencing of the four domains: *Access* is necessary for increasing *Participation*; [441] both Access and Participation provide greater Mobility and higher levels of *Adherence*; high Adherence to physical activity and exercise results in improved Performance and increased *Health and Function*, which ultimately leads to greater health protection, better self-management of health and improved quality of life.

Technology provides multiple new approaches to addressing these issues. With the growing number of technologies becoming less expensive and more ubiquitous, a unique opportunity exists for rehabilitation professionals, engineers, exercise physiologists, scientists, researchers, practitioners, and manufacturers to optimize the health and wellbeing of people with disabilities using existing and new technologies. The research and development efforts needed to break the cycle of deconditioning and promote greater health and function among people with disabilities must be considered in a logical, interdependent framework that recognizes the basic requirements for promoting physical activity among all populations, but with particular relevance to people with disabilities: increasing *access,* encouraging regular *participation*, fostering *adherence,* and improving *health and function*. New and emerging technologies must address these requirements across a range of settings that includes the home, fitness facility, and outdoor community. Whether seen as revolutionary or evolutionary, technology is certain to eliminate many of the barriers that people with disabilities are exposed to when trying to lead a physically active lifestyle. RecTech is filling the health/wellness gap that is currently not being funded by any other federal agency and addresses this important area of research and development.

#### Rehabilitation robotics

##### Need and rationale

The concept of exploring robotic devices for use by people with a disability was an early part of the RERC program. Restoring individuals’ movement ability could be accomplished either by *assisting* activities, *substituting* function through prosthetics and orthotics, or *treating* or providing therapy. The earliest stages of the RERC robotics program involved chiefly the first of these goals, and was centered at the DuPont Children’s Hospital in Wilmington Delaware.

Beginning in 2002, the *Machines Assisting Recovery from Stroke* (MARS) Rehabilitation Robotics Center has focused on developing robotics devices for restoring movement ability for people with disability through physical rehabilitation. At the time there were few commercial robotic devices designed to assist in providing physical and occupational therapy, and optimal design features were largely unexplored. Yet many people with a disability receive physical and occupation therapy, including individuals with a stroke (7M adults in the US [442]) spinal cord injury (1.3M [443]), cerebral palsy (0.8 M individuals [444], traumatic brain injury (5.3M [445]), and the elderly (projected to grow to 53M by 2020). Hence, MARS began developing systems and gathering new knowledge for manipulation and ambulation, in the pursuit of more optimal and widely accessible rehabilitation therapy tools.

Several principles drove and continue to drive the MARS vision of robot-assisted therapy. First, basic and clinical research have increasingly indicated that there is substantial potential for experience-dependent neural plasticity following neurologic injury, where change can be induced by practice [446, 447]. Second, intensive task-specific, challenge-based practice has been shown to promote the neural plasticity beneficial for recovery [448]. Yet, despite the critical need for repetitive skill practice, healthcare expenditure cuts have increasingly limited therapy time in the US and other countries. MARS has taken a key role in showing that repetitive and skillful practice can be delivered readily and effectively by appropriately designed and well-programmed robots [449–451].

Forthcoming pharmacologic, cell-based, and neural stimulation treatments have also been shown to increase plasticity and thus continue to elevate the importance of developing robotics and information technology for effective, targeted, quantifiable rehabilitation therapy [452]. Furthermore, with improvements in weight, wearability, power, sensors, actuators, computers, and energy sources, devices providing movement assistance are becoming increasingly feasible. MARS thus now seeks to develop and test robotics technology that seamlessly meets a continuum of needs, from complete movement assistance, to enhanced mobility, to therapy, to challenging exercise, to better participation in the workforce, recreation and leisure activity.

##### Advances

**Fig. 14:**
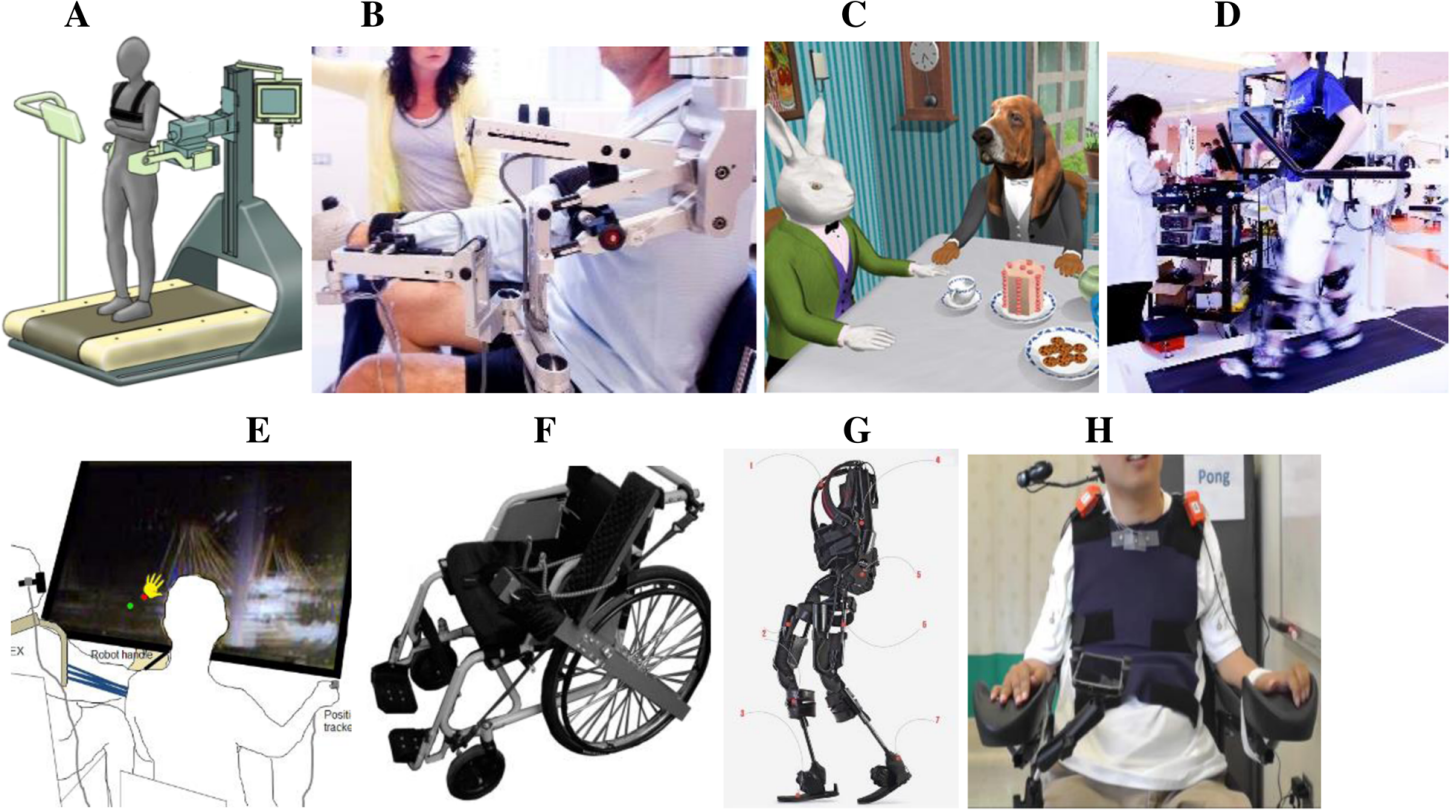
Some MARS RERC projects. **a**) The KineAssist MX® Gait and Balance Device **b**) The Armeo Spring® reaching assistance device **c**) The March Hare virtual reality therapy game **d**) The Lokomat® gait assistance robot **e**) Robotic Error Augmentation between the therapist and patient **f**) lever drive wheelchair **g**) Ekso® exoskeleton **h**) Body-machine interface for device control

MARS research has led to commercially available products that are used worldwide by individuals with disability, made discoveries that have strongly influenced the design of other products, and is continuously generating novel, promising prototypes (Fig. 14).

Development of the T-WREX arm exoskeleton [450] led to the most widely used arm exoskeleton for upper extremity rehabilitation training, the ArmeoSpring, sold by Hocoma, now in use in over 700 hospitals and clinics. This device was based partly on the WREX exoskeleton, a commercially available assistive device for children with arm weakness developed in an earlier RERC [453]. ArmeoSpring combines spring-based arm support, sensitive grip force detection, and virtual reality (VR) based games and was found to be more effective and more motivating than conventional training in rehabilitation of the upper extremity for individuals with chronic stroke.

Another recent commercial product strongly influenced by MARS RERC research is the KineAssist MX, commercialized by HDT Robotics, which uses a force-sensing, pelvic support mechanism to sense the user’s intended walking speed and direction to drive a moving surface, thus allowing a person to move at their own intended speed and pace [454]. The device is sensitive enough to allow sudden starting and stopping movements, so that dynamic balance tasks and responses to sudden disturbances can be accommodated, allowing rehabilitation therapists to safely enhance challenge in gait and balance training. MARS research was influential in improving the design of robotic gait training systems, including the Lokomat system (sold by Hocoma A.G., Zurich), by demonstrating the importance of effort and variability during gait training [455, 456]. Hocoma also licensed and commercialized patient-cooperative training regimes for the Lokomat that MARS developed.

MARS Research has also advanced the state of knowledge and changed clinical practice. MARS researchers developed the family of approaches that augment error to enhance the dynamics of learning [449, 457, 458]. MARS research produced some of the first wearable exoskeletons for the hand [459, 460] and identified the cardinal features of impaired hand control after stroke [461]. Recently, extensive clinical testing with the Ekso® and ReWalk® exoskeleton led to the development of first-of-their kind clinical evaluation and training strategies that enable individuals with paraplegia to ambulate independently at home and in the community using exoskeletons. Additionally, MARS research is initiating collaborative work on a third EMG-driven exoskeleton HAL® by Cyberdyne (Japan).

MARS clinical research continues to lead to first-of-their-kind prototypes with commercial potential. MARS research led to the first “body-machine interface”, which effectively transforms the upper-body into an adaptable joystick or keyboard controller, providing its users with the ability to control assistive devices such as wheelchairs, computers and, more recently, robotic arms [462]. Furthermore, this body-machine interface provides a means to combine in a single framework assistive functions with physical activities aimed at supporting health and promoting motor recovery. MARS researchers recently demonstrated significant clinical benefits from the first multi-user virtual environment for rehabilitation of upper extremity motor control after stroke [457]. Another project is developing a unique wearable assistive device for fall prevention that can be worn as a backpack, and that uses controlled *moment gyroscopes* that apply balance-assisting moments [463]. Recently, a novel lever drive wheelchair developed by MARS researchers allowed individuals with severe hemiparesis after stroke to propel the chair bimanually for the first time [464]. This chair is intended to increase arm activity after stroke, replacing the tens of thousands of conventional wheelchairs in stroke rehabilitation units that promote non-use of the affected arm.

##### Future directions

High cost and technological complexity make many existing upper and lower extremity robotic therapy products inaccessible for most rehabilitation clinics. The MARS team sees a strong need for devices appropriate for smaller clinics, community centers, and the home. As acceptance and price improve, we expect some devices and ideas to become well accepted.

Growth of the field is also dependent upon determination of how best to employ the robots to facilitate rehabilitation. While rigorous clinical tests, including ones performed by our center, show that robotic therapy can be measurably as effective or more effective than conventional therapy or even an expert therapist working closely with a patient [450, 457, 465], other influential studies performed by the MARS RERC show that the wrong type of robotic therapy systems, particularly in gait training, will underperform [455]. Thus, there is a clear need to develop and test algorithms, based on principles from the motor learning and neuroplasticity literature, which utilize robots in a challenging manner so as to optimize rehabilitation.

While recent emphasis has been placed upon the use of robots to improve motor control through training, the MARS group also realizes the great need for technologies that can *assist* [466]*,* as millions of individuals still cannot pursue all of the activities as fully as they wish. Consequently, the MARS family of therapeutic devices are naturally gaining assistive capabilities such as robustness, simplicity, wearability, and intelligent, task-oriented assistance. An exciting synergy is emerging between therapeutic and assistive devices.

#### Optimizing Participation through Technology

##### Needs and rationale

The overarching objective of this RERC was to Optimize Participation through Technology (OPTT). We sought to enhance the lives of individuals aging with and into disability by advancing knowledge regarding ways to maintain, restore, and enhance the sensorimotor processes that maximizes participation and quality of life; by advancing knowledge about optimal design and use of immersive technologies such as VR and video game applications for rehabilitation; by increasing capacity to conduct interdisciplinary rehabilitation research in the nexus area of disability, aging, and technology; and by improving clinical practice through effective use of our technology in the clinic and home environment.

It is well known that the probability for acquired disability increases with age [467]. Accordingly, the number of middle aged and older adults living with disabilities will grow significantly as the US population ages rapidly [468]. For those middle-aged and older adults who are living with life-long and long-term disabilities acquired at birth (e.g. cerebral palsy) or at an early age (e.g. spinal cord injury) and those who acquire their disabilities for the first time later in life (e.g. stroke, osteoarthritis), preserving health and meaningful activities throughout the lifespan is critical for living independently in the community [390, 469, 470]. Technological developments in interactive immersive technologies provide the opportunity for new strategies and interventions to enhance quality of life for people aging with disabilities and chronic conditions that affect function and mobility [471–473].

At the onset, the technologies we considered were designed to be integrated with specific programs of sensorimotor training and exercise for improving functional independence in the home and community. This approach is unique, and stands in sharp contrast to the more conventional use of technologies such as a device that can be used as a stand-alone assist for a specific task such as walking (e.g., a cane or walker). We saw the promises of leveraging innovative and integrated VR programs that combined the technology with evidence-based behavioral approaches such as muscle-specific exercises (e.g. shoulder strengthening [474, 475]) or sophisticated task-specific training protocols (e.g. hand function [476], gait and balance [476]) which harness the benefits of meaningful task practice [477] for sustained improvements in function and thereby foster participation in home, work and community life. Previous efforts in both the clinic-based and telerehabilitation domains used expensive robotic force feedback systems and/or advanced, high cost magnetic or optical sensing systems to track and capture motion data accurately at a high sampling rate, for use in the process of rehabilitation [478–481]. Such systems are typically employed in research centers and clinical settings that have sufficient economic resources. However, these high-end systems did not meet cost and deployability requirements for widespread access and adoption of home-based systems. During this time, off the shelf game consoles, such as the *Sony PlayStation® 2 EyeToy* ^*TM*^ [482, 483] and *Nintendo® Wii* ^*TM*^ [484] became available. However, these applications were either too difficult for people with disabilities to use as a therapy tool or could not be accessed or altered to improve usability. In addition to the limited options for the systematic control of stimulus parameters needed to customize interaction challenges to the needs of the user, they provided limited capacity for the recording of meaningful performance data for research purposes.

##### Advances

**Fig. 15:**
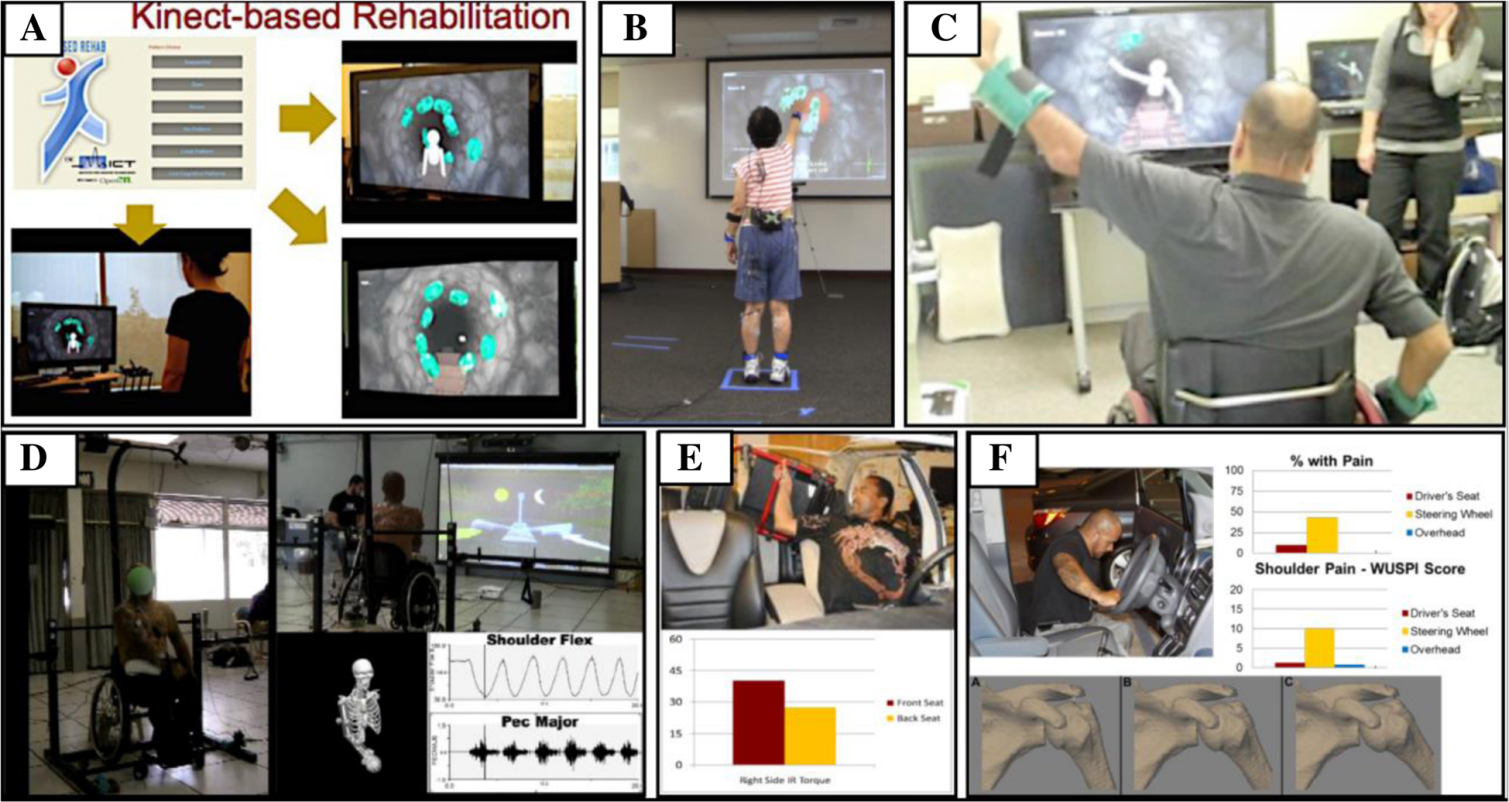
The RERC to Optimize Participation through Technology developed **a**) the FAAST open-source middleware framework to allow interface between markerless tracking technology and freely available games and Internet applications, **b**) a suite of VR-based rehabilitation games, including games for balance training and, **c**) shoulder exercises and the infrastructure to allow quantification and examination of the biomechanical variables during VR-based exercise game play. **d**) Using real-time monitoring of muscle activity, kinetics and kinematics of car transfers in those with spinal cord injury, we identified that **e**) placing the right hand on the steering wheel during the body lift portion of the transfer was associated with greater risk of developing shoulder pain than placing the hand on the driver’s seat. **f**) Routine placement of the wheelchair in the back seat was associated with reduced strength in the internal rotators of the right arm suggesting a stretch-induced injury.

Interactive video games, home entertainment and mobile technologies, and movement tracking sensors have become much more accessible and widespread, thereby providing an opportunity to advance the medical and rehabilitation health field through physical activity and participation in more engaging and enjoyable activities [485]. This was made possible through the availability and accessibility of off-the-shelf sensing technologies (i.e. Microsoft Kinect), web-enabled tools and mobile devices along with low-cost game development software applications. Our team was the vanguard in the application of such wireless body tracking for VR-based exercise and rehabilitation [486–489] (Fig. 15). We developed an open-source middleware framework (called FAAST) to allow interface between markerless tracking technology and freely available games and Internet applications (Fig. 15a) [490]. We developed a suite of VR-based rehabilitation games, including games for balance training (Fig. 15b) [491] and shoulder exercises (Fig. 15c) [492] and the infrastructure to allow quantification and examination of the biomechanical variables during VR-based exercise game play (Fig. 15d) [493, 494]. Using low-cost sensor systems, our research on shoulder pain prevention in aging wheelchair users impacted practice and policy [495]. Using real-time monitoring of muscle activity, kinetics and kinematics of car transfers in those with spinal cord injury, we identified that placing the right hand on the steering wheel during the body lift portion of the transfer was associated with greater risk of developing shoulder pain than placing the hand on the driver’s seat (Fig. 15e). We also identified that routine placement of the wheelchair in the back seat was associated with reduced strength in the internal rotators of the right arm suggesting a stretch-induced injury (Fig. 15f). This information has been included in our educational materials on car transfer [496]. We were asked by a Clinician Task Force to write a letter of testimony based on our research to the U.S. Department of Health & Human Services Centers for Medicare Services (CMS) in support of maintaining coding and funding for wheelchairs with custom-measured features [495].

##### Future directions

To strengthen the foundations of rehabilitation interventions and the interface of rehabilitation and technology, the effort must be focused toward both theoretically and empirically based principles to inform and enhance integrated interactive technologies and clinician-delivered rehabilitation solutions in collaboration with diverse end-users and professionals who have scientific, clinical, and engineering expertise [471–473]. Only through these principle driven and interdisciplinary approaches can we advance and strengthen the foundations of rehabilitation interventions and the seamless interface of rehabilitation and technology [473].

#### Telerehabilitation

##### Needs and rationale

The telerehabilitation engineering challenge concerns the application of information and communication technology to the provision of rehabilitation services to people with disabilities. It was an idea born at NIDRR in the late 1990’s. The first RERC on telerehabilitation was awarded to Catholic University and the National Rehabilitation Hospital in 1999 then to the University of Pittsburgh in 2004 and 2009. From its inception through the present day, the goals of developing telerehabilitation technology are increased access to service, more efficient use of resources, and improved rehabilitation services. In 1999 the fundamental barrier to providing rehabilitation service to a distant client was effective communication across the geographical divide. The leaders of the first RERC focused their development efforts on adapting and demonstrating video conferencing systems that used dedicated telephone lines. Their vision was much larger and included use of the Internet, telehomecare, telemonitoring and teletherapy [497]. But timing is everything and the application of many of these visionary applications were hampered by slow computer systems, slow and expensive dedicated communication channels, low-resolution video images, and a lack of availability of adequate communication channels.

##### Advances

**Fig. 16:**
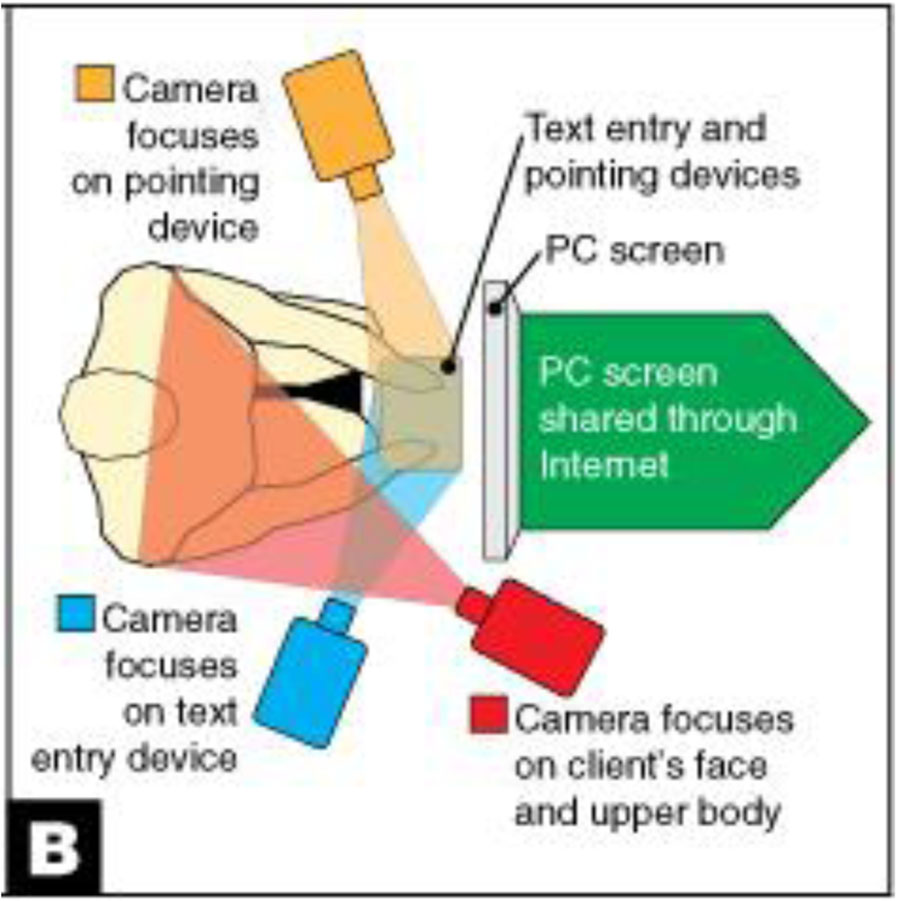
Using telerehabilitation to evaluate input devices for individuals with a disability. Remote team members interact with the client and local team members through multiple cameras, focused on the client, the client’s activation site(s) and input device(s)

Beginning 2004 the RERC on telerehabilitation at the University of Pittsburgh shifted efforts toward harnessing the emerging power of the Internet and away from dedicated communication lines. The promise of the Internet was to break down the barrier of cost and access to the critically needed communication channels for telerehabilitation in a package that was easy to use and implement but with high levels of security for privacy. The RERC began developing what evolved into the VISYTER (Versatile and Integrated System for Telerehabilitation) system [498] (Fig. 16). VISYTER is a software platform for developing telerehabilitation applications. It was designed for use in a range of environments, bandwidths and situations. For example, it included high-quality video conferencing with multiple remotely controllable cameras per site capability, integration with electronic health records, remote control of the display screen, heads-up teleprompter and flexible hooks for rehabilitation application specific needs such as stimuli presentation. The RERC used VISYTER for several applications including remote wheelchair prescription [499], autism assessment [500, 501], and a self-management program for persons with chronic lower limb swelling and mobility limitations [502] among others. Presently, this system has been adopted within the state vocational rehabilitation program within Pennsylvania (Office of Vocational rehabilitation) as a tool for extending the reach of vocational rehabilitation counselors to rehabilitation customers within their state-operated vocational rehabilitation facility (Hiram G. Andrews Center in Johnstown, PA), for consultation among rehabilitation professionals across the state, to supervise staff and students, and to conduct remote psychological and neuropsychological assessments.

As mobile devices with Internet capability became available, the RERC on Telerehabilitation worked to develop and evaluate a telerehabilitation platform and applications that took advantage of their capabilities, specifically, applications that benefited from persistent long-term interaction between the client and the care provider [503]. The platform development work resulted in a system called iMHere, a mobile health platform focused on supporting self-care in the management of chronic conditions [504]. As an example demonstrating what rehabilitation needs can be satisfied with mobile technologies, the RERC developed and implemented a wellness program for people with Spina Bifida [505].

##### Future directions

Now that mobile devices with reliable and continuous Internet connections are commonplace, our community of rehabilitation scientists and engineers has the opportunity to develop systems and applications on top of these platforms that can have a potentially large and profound impact on the effectiveness of rehabilitation strategies. In many cases, the ever-present mobile devices allow for interventions that were not previously possible. State of the art and best practice in rehabilitation service delivery has decidedly acknowledged the importance of the individual’s natural context into its conceptualization of disability, but only limited efforts have focused on systematically integrating the natural context into assessment and intervention. Techniques such as Ecological Momentary Assessment and Ecological Momentary Intervention can provide contextually relevant “rehabilitation in the real world” to persons with greatest need, at the time and in the context where rehabilitation is most likely to make an impact – the natural or lived-in environment. To accomplish this vision, development and evaluation of new ecological momentary rehabilitation tools, techniques and innovations that can be merged with evidence-based practices of clinical in rehabilitation is needed. Recent telerehabilitation technological advances and mainstream information technology may be enabler for implementation of this vision.

So the past, present and future of telerehabilitation will likely continue to follow mainstream information and communication technology evolution. The RERC on telerehabilitation started in the 90’s when communication links between institutions allowed people with disabilities at one clinic to communicate with and receive service from a provider at another clinic. As the Internet grew in the early 2000’s and higher capacity broadband connections became available in peoples homes, schools and workplaces, new possibilities for rehabilitation services were developed and evaluated. Next, people began carrying powerful mobile devices with fast processors with them as they went about their daily lives and even more possibilities for improved rehabilitation service emerged. Now, mobile devices attached to our bodies in the form of watches and wristbands may provide the next level opportunity. This is the current technology in need of RERC attention. What’s next? Will communication devices inside of our bodies become commonplace? Time will tell. But whatever that next level of communication may be, a rehabilitation engineering research center will be needed to explore its application for improving the lives of people with disabilities.

#### Timing investigation dosage implementation

##### Need and rationale

The United States spends more on healthcare than any other industrialized nation [506]. In 2013 healthcare expenditures, including federal and private insurance, accounted for more than 17% of the Gross National Product ($2.9 trillion) and are projected to be close to 19% by 2023 ($5 trillion) [507]. Until recently, healthcare reimbursement was based on a cost-reimbursement, charge-basis system and billing was related directly to the number of days hospitalized and the number of tests and services received. More was considered to be better. To slow the growth of healthcare expenditures, the Centers for Medicare and Medicaid Services and other health insurance providers have imposed increasingly restrictive reimbursement guidelines. In 1982 a prospective payment system was instituted for acute inpatient care based on diagnosis-related groups. In 1997 Congress passed the Balanced Budget Amendment which put all components of post-acute care, including inpatient rehabilitation facilities and outpatient therapy, under a prospective payment system. The end result has been significantly decreased lengths of acute and rehabilitation hospitalization and fewer sessions of outpatient therapy available to patients.

These constraints in rehabilitation healthcare are playing out at the same time when the demand for rehabilitation services is expected to increase. We are getting older as a country with the number of people age 65 and over expected to increase from 40.3 million in 2010 to more than 80 million by 2050 [508]. The increasing incidence of stroke, Parkinson’s, arthritis and other chronic diseases associated with an aging population are expected place additional demands on the healthcare system. Providing quality care while reducing per-patient costs requires implementing innovative evidence-based strategies.

##### Advances

There is growing empirical evidence for the effectiveness of repetitive “task-specific” training in rehabilitation and for neural plastic changes following task-oriented training[509–512]. A key factor in neural recovery appears to be the availability of intensive, long lasting, and repetitive practice [509, 513, 514]. Motivated by these requirements, there is increasing interest in the development of robotic devices for task-oriented therapy of upper and lower extremities for patients with neurologic injuries [515–518].

A critical aspect to the clinical success of any therapeutic device is determining how the device could potentially be used to benefit both patients and therapists. For those patients who can tolerate additional exercise, a robotic therapy device could be used relatively unsupervised as an adjunct to regular therapy. Another possibility is for therapists to integrate the device into their regular therapy. This could improve the effectiveness of therapists by providing them a tool that reduces the routine manual manipulation aspects of their treatment, thereby allowing them to focus on other aspects of therapy.

What has been largely ignored so far is the question of therapy distribution in time. We usually deliver care on a regular basis with sessions equally spaced in time, and the total number of sessions is usually determined by insurance or other external factors, such as therapist scheduling, family constraints, and other factors, but not by the specific needs of the patient, or on a rational evidence-based approach.

In October 2013 the Rehabilitation Engineering Research Center on Timing Investigation Dosage Implementation (TIDI) was funded to develop a center designed to establish a rational basis for choosing the appropriate time distribution for use of robotic and computer based interventions in rehabilitation therapy.

During the first two years of the project, investigators will measure the response to single therapy episodes, tracking the time-course of task acquisition (the learning phase), and most importantly, the time course of decay (forgetting). These data will then be used to model the time courses of repeated therapy and to quantify the learning/forgetting process. We can also determine whether summation of learning is linear, amplified, or reduced – all key factors in planning therapy schedules. These models will then be tested during the later stages of the program.

**Fig. 17:**
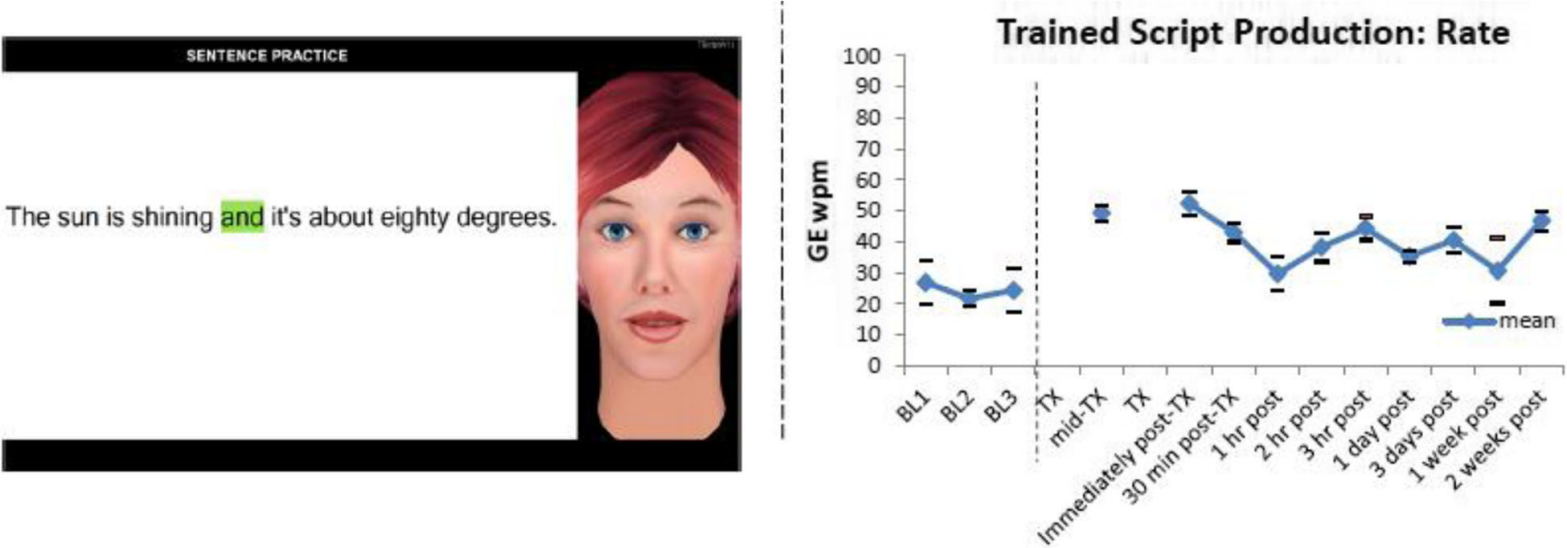
Learning and forgetting during script training in aphasia. Script training is provided for one hour. Left: Screenshot of the computer-based script training that is delivered by a virtual therapist. Right: Learning - Performance increased from a baseline mean rate of 24.3 words per minute (WPM) to 52.3 WPM immediately post-treatment. Forgetting – Performance decreased to 35.3 WPM at 1 day and 30.65 WPM at 1 week post treatment

The areas of study for the RERC include an investigation of mixed-reality rehabilitation. This is a novel system that allows people with stroke to perform naturalistic, repetitive practice of everyday movements, while receiving intuitive and useful visual, auditory, and eventually haptic feedback related to key aspects of the voluntary movement. We also are developing computer based algorithms for restoration of language after stroke using a computer-based treatment program, *AphasiaScripts* [519] (Fig. 17). A third area of investigation involves the optimal timing of lower extremity therapy using a portable ankle rehabilitation robot that conducts active and passive stretching. Lastly, we are examining the timing of training for use of a walking exoskeleton in spinal cord injured persons.

##### Future directions

In the future we can expect that aggressive steps by insurers and employers, new models of healthcare delivery and the focus by the Affordable Care Act on healthcare value and patient outcomes will continue to exert downward pressure on healthcare providers. Healthcare providers are seeking better evidence on which to base decisions for healthcare delivery. For example, a 2013 Medicare Payment Advisory Commission report to Congress noted [520] *“The Medicare program currently lacks clear clinical guidelines as to who needs outpatient therapy, how much therapy they should receive, and how long they need services.”* Understanding the time course of the effects of rehabilitation therapies will care to be more individualized, more effective, streamlined, and dynamic.

## Conclusions

By reviewing rehabilitation engineering research progress through the lens of NIDILRRs’ RERCs, our goal was to gain insight into the evolving nature and demands of rehabilitation technology development, as well as the role of a multidisciplinary structure, like the RERCs, in shaping the production of this technology. Our assessment is that the NIDILRR RERC program is indeed highly multidisciplinary, and this approach helped diversify the scope and alter the perspective of rehabilitation engineering research in four key ways.

*Theme 1: Diversification of disabilities addressed*

Since the inception of the RERCs in the 1970’s, the scope of rehabilitation research has expanded to address different types of disabilities and the ever-increasing complexity in the interaction between the person and the environment. Most of the work at the original Rehabilitation Engineering Centers funded in the 1970’s focused on technologies for individual mobility, such as prosthetics, functional electrical stimulation, and control systems for powered wheelchairs. While this line of work is important and is advancing right now in exciting ways, the scope of mobility research increased to include both technologies and policies that address mobility and other needs at a societal level. A prime example is the Tiramisu mobile application that has been used by thousands to access public transit information. RERC work has also expanded to address issues related to vision and hearing function, as well as the complex spectrum of cognitive impairments. Another trend is that RERC research has expanded into issues of rehabilitation therapy and exercise, including learning and plasticity, which are powerful modulators of health and impairment status (as exemplified by the five RERCs focused on Rehabilitation Therapy and Exercise – see Table 1).

### Theme 2: From specialized to mainstreamed technologies

Another change of perspective has been to increasingly understand that people with a disability are integrated with people without a disability into an interdependent society through mutually-usable technology, via, for example, the internet, wireless technologies, and architecture. A growing emphasis on universal design has brought a cross-disability perspective to design of rehabilitation technology and a concern for broader issues of usability, recognizing that people with disabilities have similar concerns for durability, reliability, and quality of the customer service as the broader population.Increasingly, through universal design, advancements in rehabilitation technology are becoming mainstream and benefitting broader populations. Universal design is now used in many of the RERCs to support not only social participation of people with disabilities, but others as well. For example, using the Tiramisu mobile application as an example again, many users without a disability are unaware that their crowdsource contributions are specifically designed to help riders with disabilities. The knowledge of the rehabilitation technology community has been applied in mainstream design in many ways; examples include curb ramps as a universal feature of sidewalks, captioning as a universal feature of television, the incorporation of accessibility features in computer operating systems, voice over for making gesture based computing accessible, touch responsive models for wayfinding, and standards for basic accessibility to all homes. RERCs have played a role in facilitating the adoption of these innovations.

### Theme 3: Expanding use of information technologies

A major evolution in terms of technology focus of the RERC program since the 1970’s is the greatly increased use and development of information technologies, including the internet, miniaturized sensors, portable computing, mobile apps, and virtual reality. Most of the RERCs currently emphasize these sorts of technological approaches, and there have been successes related to these technologies (Table 2). One might say that information technologies are the substrate on which rehabilitation innovation has recently flourished. In a double-edged sword, however, the move by society to digital technologies, everywhere, in every activity not only enables innovative solutions, but creates profound new issues, particularly with respect to participation. Such complex, bidirectional effects demand insight and creativity from rehabilitation engineering.

### Theme 4: Multidisciplinarity as a contributor to rehabilitation engineering

As stated in the Introduction, the first objective of the nascent REC’s, defined at a meeting held by the National Academy of Sciences in 1970, was “to improve the quality of life of the physically handicapped through a total approach to rehabilitation, combining medicine, engineering, and related science.” The RERC’s have attempted to meet this objective, and, in fact, the scope of “rehabilitation” covered by the RERC’s is even broader than at first implementation, including more types of disabilities (Theme 1 above). The knowledge of the rehabilitation technology community has been applied in mainstream design in several ways (Theme 2 above). Involvement of “medicine” has expanded to include the fields of therapy, exercise, prosthetics and orthotics, and psychology, and “science” now includes, for example, computer and information science in ways not envisioned (Theme 3 above). Researchers in a broad range of fields (36 total fields reported, see Table 3), including about 11% of the staff who have a disability related to the project on which they are working, have collaborated to produce many impactful products (see Table 2). However, growth possibilities still exist, as we describe next.

### Growth Opportunities and Possible Future Directions

Currently, 70% of the research and development staff of RERCs are in engineering fields, 23% in clinical fields, and only 7% come from basic science fields (Table 3). Does this distribution adequately implement the “total approach” desired of the RERC structure in its original charter? When asked to critique the RERC program as we collaborated on writing this paper, some RERC directors advocated that science and scholarship increasingly drive program activities. Therefore, strengthening the involvement of scientists, and the use of science to drive activities, is one potential growth opportunity for the RERC program. Several directors thought that the review process could be modified to increase scientific rigor, in part by increasing the size of and expertise on the review panels, and in part by revising the rubrics used to evaluate applications. Other directors pointed out, however, that while a good RERC will advance science and use science, the primary goal is not science but the creation of solutions using science, technology, and engineering. Arguably, it this pragmatic emphasis that makes the RERC program unique and important compared to many other funding programs aimed at rehabilitation research, and perhaps accounts for the high involvement of engineers.

The future success of the RERC program will also likely depend on incorporating new technological approaches. We observed above that one of the major evolutions in the scope of rehabilitation engineering research within the RERC program was the increasing focus on information technologies. In the near future, revolutions in additive manufacturing (e.g., 3D printing), biomaterials, artificial intelligence and robotics (e.g. deep learning and self-driving cars), as well as neural interfaces (e.g. brain computer interfaces) and regenerative medicine (e.g. stem cell therapy coupled with rehabilitation) have the potential to reshape the portfolio of technologies used by rehabilitation engineering. How will the RERC program respond and lead as new technologies come to bear on key, persistent needs of individuals with a disability? Some directors advocated for the use of “open” rather than “targeted” RERC calls, because they believe they allow for greater creativity and new ideas.

Encouragingly, about 11% of the research and development staff of RERCs have a disability that informs their work. However, the directors agreed that individuals with a disability could be even more frequently and more intimately involved in the design process, both as consumers and designers. In addition, they see a growth potential for interaction between RERCs (recall only 13 of 19 RERCs reported some level of interaction with each other), and also with industry. Some directors noted that there is a need to identify cross-cutting concepts (examples given included fundamental psychological needs, the role of self-management to promote core goals, consideration of the timing of rehabilitation interventions, and the consistent incorporation of universal design) to improve and focus collaboration. The future success of rehabilitation engineering will likely depend on the continued expansion of multidisciplinary collaboration, inclusion of new expertise and new users, increasing participation of individuals with disabilities, and identification of innovative themes that promote new ways of looking at key problems and opportunities.

## Abbreviations

AAC: Augmentative and Alternative Communication
ADA: American’s with Disabilities Act
AVE^2^ D: Advanced Virtual Environment Exercise Device
AIMS: Activity Inclusion Mapping System
ANSI: American National Standards Institute
APT: Accessible Public Transportation
ASTM: American Section of the International Association for Testing Materials
CEAL: Challenging Environment Assessment Lab
CNC: Computer Numerical Control
CAN: Consumer Advisory Network
CP: Cerebral Palsy
GIS: Geographical Information Systems
ISO: International Organization for Standardization
KEI: Keyboard Emulating Interface
MAG: Mobile Accessibility Guide
MARS: Machines Assisting Recovery from Stroke
OI: Osteogenesis Imperfecta
P&O: Prosthetics and Orthotics
NDC: neurodevelopmental condition
NHTSA: National Highway Traffic Safety Administration
NIHR: National Institute on Handicapped Research
NIDILRR: National Institute on Disability, Independent Living, and Rehabilitation Research
OPTT: Optimize Participation through Technology
POWERS: Personalized Online Weight and Exercise Response System
REC: Rehabilitation Engineering Center
RERC: Rehabilitation Engineering Research Centers
SUN: Survey of User Needs
TBI: traumatic Brain Injury
TEAMM: Technologies to Advance Manipulation and Mobility
TExT-ME: Telehealth Exercise Training for Monitoring and Evaluation
TMAP: Tactile Map Automated Production
TDD: Telecommunications Device for the Deaf
TIDI: Timing Investigation Dosage Implementation
TIKTOC: Technology Increasing Knowledge: Technology Optimizing Choice
TTAM: Trace Transparent Access Module
TTY: Text Telephone
T-WREX: Therapy Wilmington Robotic Exoskeleton
RESNA: Rehabilitation Engineering Society of North America
UD: Universal Design
UMTRI: University of Michigan Transportation Research Institute
VEE: Virtual Exercise Environment
VEP: Visual Evoked Potential
VR: Virtual Reality
WTS: Wheelchair Transportation Safety
WTORS: Wheelchair Tiedown and Occupant Restraint Systems
